# From Flue Gas to Syngas: Composite Electrode Based on Ionic Liquid and Microporous Polymer for MEA‐Based CO_2_ Electrolysis

**DOI:** 10.1002/anie.202513103

**Published:** 2025-08-14

**Authors:** Hesamoddin Rabiee, Abhijit Dutta, Penghui Yan, Lei Ge, Fatereh Dorosti, Xin Yu, Alain Rieder, Peter Broekmann

**Affiliations:** ^1^ Department of Chemistry Biochemistry and Pharmaceutical Sciences University of Bern Freiestrasse 3 Bern 3012 Switzerland; ^2^ NCCR Catalysis University of Bern Freiestrasse 3 Bern 3012 Switzerland; ^3^ School of Chemical Engineering The University of Queensland Brisbane QLD 4072 Australia; ^4^ Centre for Future Materials University of Southern Queensland Springfield QLD 4300 Australia

**Keywords:** CO_2_ Capture‐reduction integration, Electrochemical CO_2_ reduction reaction, Gas‐diffusion electrode, Ionic liquid, Polymer of Intrinsic porosity

## Abstract

The electrochemical CO_2_ reduction reaction (ECO_2_R) offers a promising pathway to convert CO_2_ into value‐added products. While catalyst advances remain crucial, gas‐diffusion electrodes (GDEs) architecture is equally vital in CO_2_ electrolyzer design. Most ECO_2_R studies use pure CO_2_ feeds, whereas industrial sources like flue gas contain ∼15% CO_2_, requiring costly purification. Eliminating this step demands electrolyzers that directly process impure streams via in situ separation. Here, we introduce a composite GDE (CGDE) featuring a thin CO_2_‐selective interlayer of intrinsically microporous polymer (PIM‐1) reinforced with the CO_2_‐philic ionic liquid [Emim][BF_4_]. This layer selectively adsorbs CO_2_ and suppresses N_2_/O_2_ existence at the catalyst interface. In simulated flue gas (15% CO_2_, 5% O_2_ in N_2_), the CGDE with 20 wt% [Emim][BF_4_]/PIM‐1 achieved >70% CO Faradaic efficiency (FE) at 100 mA cm^−^^2^, versus ∼20% FE for a pristine GDE. Multiphysics simulations confirmed effective CO_2_ delivery through the selective layer, with minimal O_2_ permeation. Cost estimation analysis indicates around 25% reduction in CO's minimum selling price using the integrated design and >50% under ideal performance metrics by eliminating compression/transport. These results demonstrate that advanced electrode design with CO_2_‐selective interlayer enables direct mixed‐gas ECO_2_R, establishes key design criteria for selective layers, and significantly improves process economics.

## Graphical abstract

A composite gas‐diffusion electrode (GDE) with a thin CO_2_‐selective interlayer beneath the catalyst facilitates direct MEA electrolysis of simulated flue gas (with O_2_) to CO/syngas, eliminating costly pre‐purification. The interlayer—an intrinsically microporous polymer scaffold infused with CO_2_‐philic [Emim][BF_4_]—delivers high CO_2_/N_2_ and CO_2_/O_2_ selectivity, directing CO_2_ to the catalyst layer, and greatly enhancing process economics.

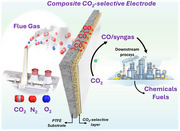

## Introduction

Electrochemical CO_2_ reduction reaction (ECO_2_R) has emerged as a promising carbon valorization approach powered by renewable electricity.^[^ ^1^ ^]^ Over the past two decades, significant progress has been made in developing electrocatalysts for ECO_2_R to target products such as CO, formate, and multi‐carbon compounds (C_2+_).^[^ ^1^, ^2^ ^]^ Notably, CO is among the most cost‐effective products, serving as an intermediate for further reduction to hydrocarbons or as a feedstock for downstream processes like Fischer–Tropsch synthesis.^[^ ^3^, ^4^ ^]^ Concurrently, electrode and electrolyzer designs, particularly gas‐diffusion electrodes (GDEs), have been intensively studied for continuous ECO_2_R operation.^[^ ^5^, ^6^, ^7^ ^]^ Efforts to overcome common GDE limitations, such as flooding,^[^ ^8^ ^]^ have led to reports of highly stable ECO_2_R at high reaction rates.^[^ ^9^, ^10^ ^]^ Furthermore, advances in electrolyzer design have enabled the production of high‐purity products at rates approaching commercial viability.^[^ ^11^, ^12^ ^]^

Despite these advancements, most studies continue to rely on pure CO_2_ feeds, whereas waste streams such as flue gas typically contain only 15%–20% CO_2_. Supplying pure CO_2_ to ECO_2_R reactors is expensive, with recent analyses indicating that CO_2_ pre‐purification constitutes a significant portion of the overall process cost.^[^ ^13^, ^14^ ^]^ To address this, efforts have focused on integrating CO_2_ capture and reduction, aiming for a more economical route to directly convert diluted CO_2_ streams.^[^ ^15^, ^16^ ^]^ A common approach involves feeding CO_2_‐rich media, either based on amines, ionic liquids, or alkaline aqueous solutions, into the electrolyzer, known as solution‐based integration.^[^ ^17^, ^18^ ^]^ However, reaction rates are often limited due to strong chemical binding between CO_2_ and the capture media (e.g., ionic liquids or amine solutions), leading to low CO_2_ utilization efficiency.^[^ ^19^ ^]^ Additionally, these capture media may not be optimal electrolytes for ECO_2_R, and electrolyte choice significantly influences reaction pathways and kinetics.^[^ ^20^, ^21^ ^]^ The solution‐based integration still comprises two steps, the CO_2_ capture unit and CO_2_ utilization unit (or solution regeneration); in other words, the diluted CO_2_ does not directly enter the electrolyzer.

An alternative integration strategy involves engineering the GDE to be CO_2_‐selective, enabling direct use of mixed gas feeds in conventional gas‐phase electrolyzers. This promising approach was outlined in our recent perspective, which proposed potential designs for reduction‐capture integration in GDEs.^[^ ^17^ ^]^ Recent studies have demonstrated that mixed gas feeds containing as little as 10% CO_2_ can be effectively utilized for syngas or ethylene production at moderate current densities.^[^ ^22^, ^23^, ^24^, ^25^, ^26^ ^]^ This was achieved either by incorporating CO_2_‐affinity agents like amines into the catalyst^[^ ^22^ ^]^ or introducing a separate layer.^[^ ^27^, ^28^ ^]^ Moreover, typical flue gas contains O_2_, whose reduction is thermodynamically more favorable than ECO_2_R, making it necessary to minimize the O_2_ availability near the catalyst layer to avoid incurring high separation costs.

In the design of CO_2_‐selective GDEs, careful engineering of the CO_2_‐philic layer is crucial to ensure sufficient CO_2_ delivery from the mixed gas stream to the catalyst layer, while avoiding transport or electrical resistance issues within the GDE. Moreover, the positioning of the selective layer is critical: placing it on the backside of the GDE serves solely as a separation barrier while positioning it beneath the catalyst layer directly influences the reaction microenvironment. Polymers of intrinsic microporosity (PIMs) have been explored as a new class of materials for gas separation applications.^[^ ^29^, ^30^ ^]^ The highly porous nature of PIM acts as CO_2_ basket or CO_2_ reservoir with high capacity to adsorb CO_2_ selectively.^[^ ^31^ ^]^ Additionally, PIMs facilitate coexistence of gas and liquid phases, promoting the formation of triple‐phase interfaces and active sites for electrochemical reactions, and stabilize the gas‐liquid microenvironment by hindering excessive bubble formation in contact with the catalyst layer.^[^ ^32^, ^33^, ^34^ ^]^ PIM‐1, the most widely studied PIM, is composed of a rigid and contorted backbone formed by fused aromatic rings and spirobisindane units. This architecture disrupts chain packing and rotation, leading to high internal free volume and permanent microporosity with typical pore sizes below 2–3 nm. The rigidity arises from non‐rotatable aryl–aryl bonds and the presence of spiro linkages, where the central tetrahedral sp^3^‐hybridized carbon enforces a nonplanar geometry. This prevents linear alignment of polymer chains, locking in free volume and enabling permanent porosity.^[^ ^35^, ^36^ ^]^

The CO_2_ affinity of high‐performance porous polymeric adsorbents can be further enhanced by integrating CO_2_‐philic ionic liquids (ILs) with high selectivity.^[^ ^37^, ^38^ ^]^ A promising candidate is [Emim][BF_4_], an imidazolium‐based IL with superior CO_2_/N_2_ and CO_2_/O_2_ selectivity compared to ammonium‐ and phosphonium‐based ILs, owing to its smaller molar volume and CO_2_‐solubility selectivity.^[^ ^39^ ^]^ Beyond gas separation, the selective layer can also influence catalytic performance by engaging directly with the catalyst layer through [Emim][BF_4_]‐CO_2_ interactions.^[^ ^19^, ^40^, ^41^ ^]^ ILs are known to mediate ECO_2_R intermediates,^[^ ^42^ ^]^ and influence reaction pathways when used as electrolyte,^[^ ^43^, ^44^ ^]^ although they face limitations in current density due to mass transfer resistance and scalability challenges. Incorporating a CO_2_‐philic IL with high ionic conductivity into a PIM layer creates a multifunctional layer in the GDE that offers: (1) high local CO_2_ availability through strong CO_2_ adsorption and CO_2_/N_2_ selectivity; (2) microenvironment modulation via the PIM's high surface area and triple‐phase interface formation; and (3) ECO_2_R mediation by both IL and the microporous structure of PIM, as evidenced by recent studies.^[^ ^19^, ^32^, ^42^, ^44^, ^45^, ^46^, ^47^, ^48^ ^]^

**Figure 1:**
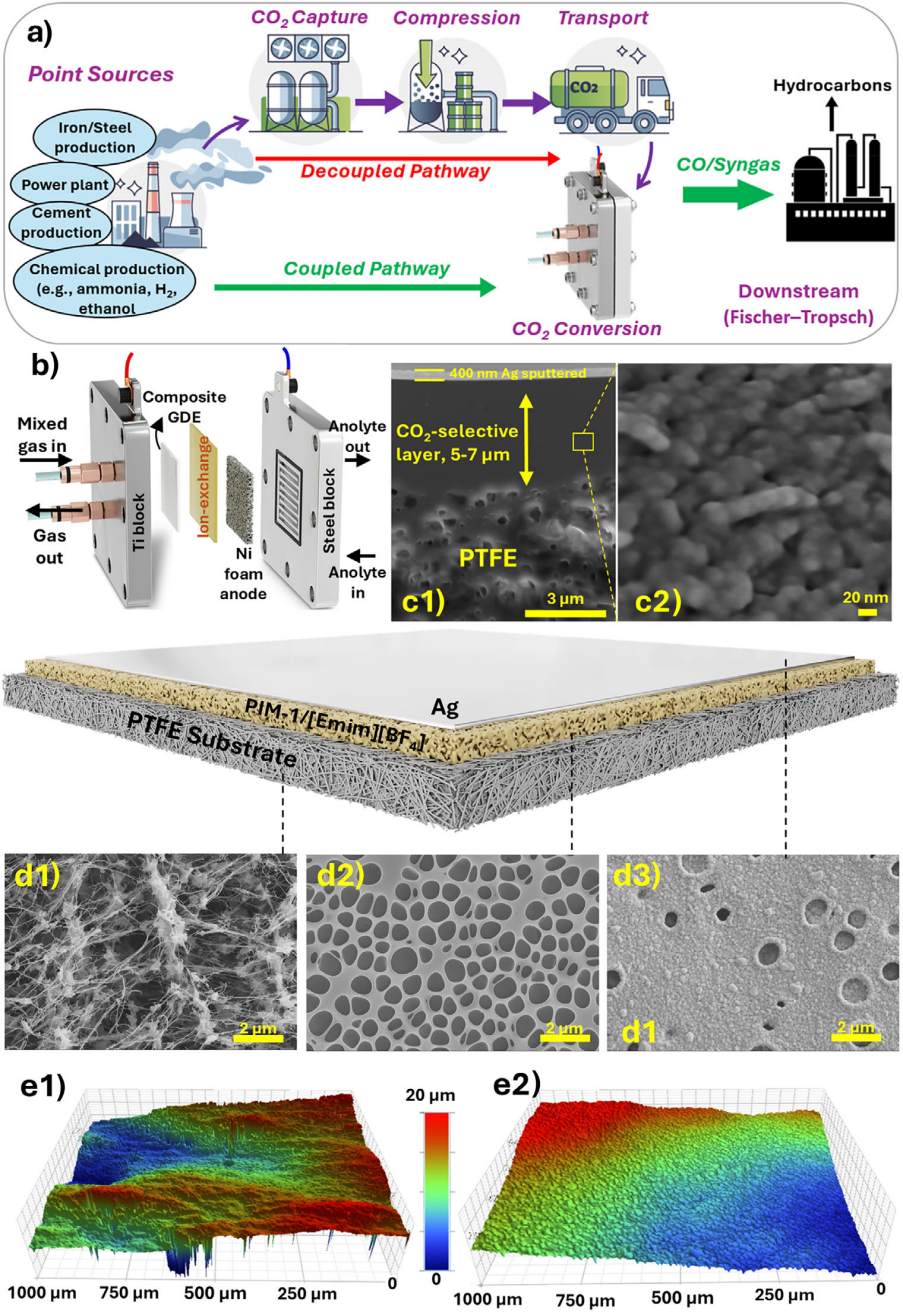
a) Schematic of the proposed GDE‐based CO_2_ capture‐reduction process, demonstrating direct feeding of less pure CO_2_ into the system via a CO_2_‐selective GDE; b) Membrane‐electrode assembly electrolyzer used in this study; c) FIB‐SEM cross‐sectional image of the composite GDE comprising a PTFE substrate, CO_2_‐selective interlayer, and Au‐sputtered layer, with high‐magnification detail of the interlayer showing its microporous structure; d) Schematic of the CGDE's three‐layer structure; surface SEM images of the PTFE substrate (d1), after interlayer coating (d2), and after Ag sputtering (d3); e) White light interferometry images of Ag‐sputtered GDEs with (e1) and without (e2) the interlayer captured with 50x magnification lens.

Herein, as illustrated in Figure 1a, we engineered a composite gas‐diffusion electrode (CGDE) featuring an efficient CO_2_‐selective layer composed of PIM‐1 and the CO_2_‐philic ionic liquid [Emim][BF_4_]. This design enables direct conversion of mixed gas feeds containing as low as 15% CO_2_ diluted in N_2_ with 5% O_2_, mimicking flue gas, into CO. By facilitating continuous CO_2_ adsorption from the mixed stream and subsequent desorption driven by concentration gradients and catalytic consumption, the CGDE effectively acts as a CO_2_ sink, potentially eliminating energy‐intensive compression and regeneration steps in conventional CO_2_ conversion. The CO_2_‐selective layer was integrated with a PTFE‐based gas‐diffusion layer sputtered with Ag as the catalyst. MEA‐type (membrane electrode assembly, Figure 1b) electrolyzer tests were conducted to evaluate the effects of the interlayer, [Emim][BF_4_] loading, and CO_2_ concentration. While the bare Ag‐sputtered GDE exhibited a significant drop in CO Faradaic efficiency under a 15% CO_2_ 5% O_2_ feed to as low as 20%, the CGDE achieved FE_CO_ of 65% due to the synergistic effects of the PIM‐1/[Emim][BF_4_] layer, providing in situ CO_2_ purification, promoting triple‐phase formation through its porous structure, and mediating CO_2_ conversion in proximity to the catalyst layer. These results underscore the importance of designing composite GDEs with tailored microstructures to enable direct utilization of dilute CO_2_ streams, thereby eliminating costly pre‐purification steps.

## Results and Discussion

### Fabrication and Characterization of Electrodes

We fabricated composite GDEs (CGDEs) with a three‐layer structure (Figure 1c), incorporating a CO_2_‐selective interlayer sandwiched between the PTFE substrate and the Ag catalyst layer. The selective layer was coated on the smooth side of the PTFE substrate, as coating on the rough polypropylene backer was unsuccessful due to surface irregularities that prevented uniform thin film formation. PIM‐1 and [Emim][BF_4_] were selected for the selective layer, with PIM‐1 providing high microporosity (Figure 1c2) as a scaffold for the CO_2_‐philic IL. Chloroform was used as the solvent for PIM‐1 due to its low flash point, facilitating rapid evaporation and yielding a porous layer (Figures 1d and S1). [Emim][BF_4_] was added at various loadings (up to 20 wt%), with higher IL concentrations causing surface aggregation, exceeding the mixing capacity with PIM‐1, and thus were excluded from further tests. Thicker selective layers were achieved by multiple coating cycles (Figure S2), but beyond 15 cycles (∼7 µm), detachment and surface defects occurred due to the glassy nature of PIM‐1. Therefore, electrolysis tests were performed with CGDEs having a 5–7 µm interlayer thickness. The surface pores observed were not pinholes but interconnected micropores that remained permeable to gases after several coating cycles to promote gas regulation, as confirmed by high‐magnification SEM imaging (Figure S3) and the presence of micropores capable of gas sieving and storage. An Ag layer (∼400 nm) was sputtered onto the top (Figure S4). The pressure‐dependent gas permeability of untreated and coated GDEs at elevated pressures indicated the coating reduces the gas permeation, but still sufficient gas could pass to the catalyst layer (Figure S5).

The PIM‐1 coating also enhanced uniform gas permeation through the PTFE substrate to the catalyst layer. Gas permeability measurements (Figure S5) revealed high permeance for the untreated PTFE substrate, likely due to its non‐uniform pore structure and potential pinholes, characteristic of membranes designed for water filtration, where the reported 0.45 µm pore size reflects solid particle filtration rather than actual pore architecture. However, the PTFE substrate coated with a PIM‐layer showed a steadier increase in gas flux at elevated pressures. This concept parallels the role of a gutter layer in thin‐film composite membranes used for gas separation, which compensates for porous substrate imperfections and provides a smooth surface for the selective layer.^[^ ^49^ ^]^ The PIM‐1 layer fulfills a similar function, regulating gas flux without impeding sufficient CO_2_ delivery to the catalyst layer. Moreover, 3D microscopic imaging of CGDEs with and without the PIM‐1 layer (Figure 1e) demonstrated a significantly smoother surface for the coated electrode, further validating the PIM‐1 layer's role in mitigating surface imperfections of the PTFE substrate.

**Figure 2:**
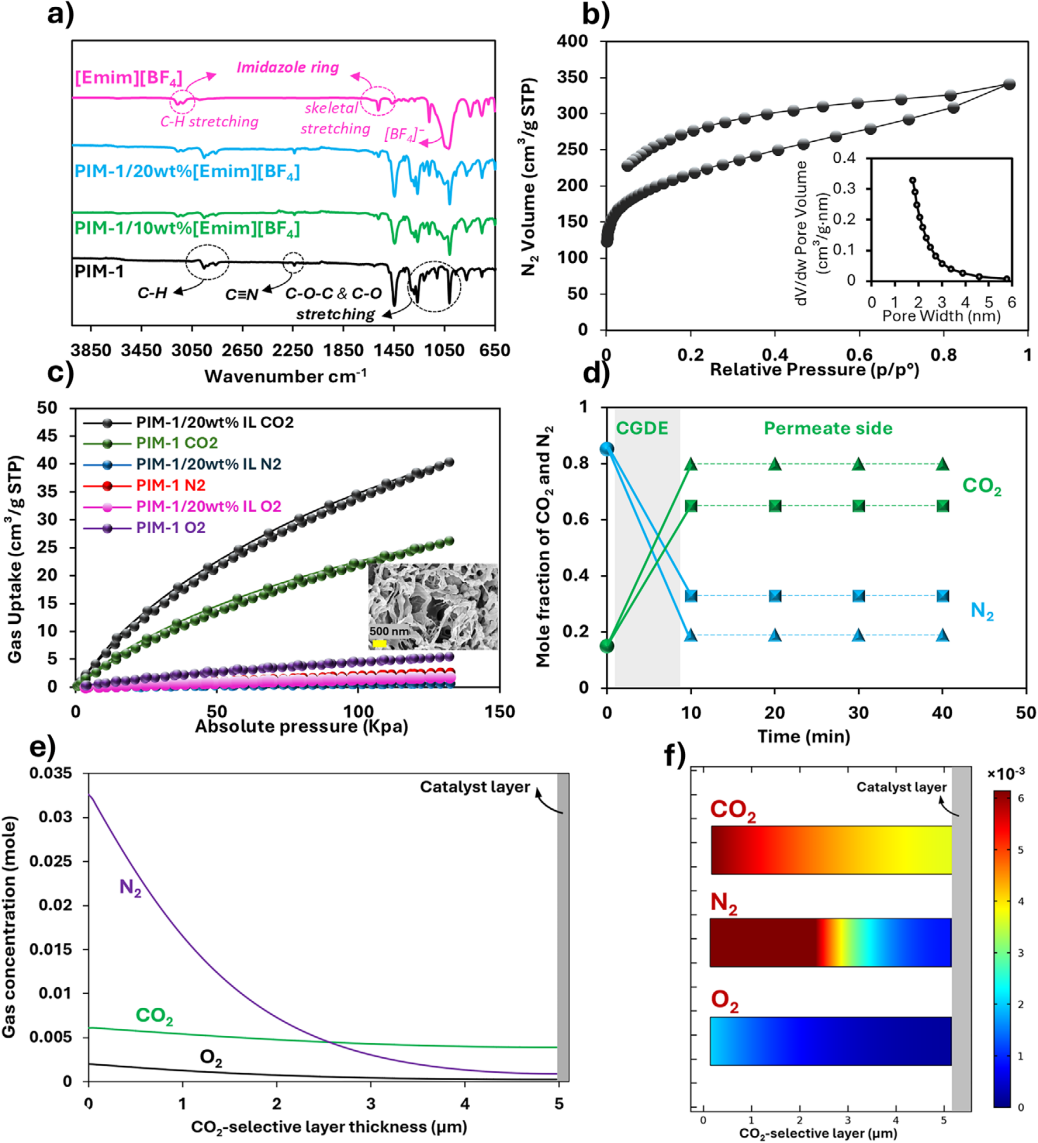
a) FTIR spectra of PIM‐1, [Emim][BF_4_], and PIM‐1/[Emim][BF_4_] mixtures with major characteristic peaks marked; b) N_2_ sorption isotherm at 77 K and pore size distribution (inset) of PIM‐1; c) CO_2_, O_2_, and N_2_ adsorption isotherms at 298 K on pure PIM‐1 and PIM‐1/20 wt% [Emim][BF_4_]; d) Breakthrough curves for CGDEs with PIM‐1 and PIM‐1/20 wt% [Emim][BF_4_] under 1.1–1.2 psi overpressure, with a mixed gas (15% CO_2_ in N_2_ balance) inlet flow rate of 30 mL min^−1^ (■ is for GDE with pure PIM‐1 layer, ▲ is for the CGDE with PIM/20 wt% [Emim][BF_4_]), The concentration (mole fraction of CO_2_ and N_2_) at *t* = 0 shows the initial inlet gas concentrations behind the CGDE, and the ones at *t* ≥ 10 min represents the gas concentrations in permeate side of the GDE; e), f) multiphysics simulation of CO_2_, N_2_, and O_2_ permeation through the interlayer and concentration changes, performed using COMSOL (details in Supporting Information. The gas concentrations in (e and f) are based on ideal gas rule and inlet concentration of 15% CO_2_, 5% O_2_, 80% N_2_ at room temperature.

FTIR spectra of the PIM‐1‐coated PTFE substrate (Figure 2a) exhibited characteristic peaks corresponding to aliphatic and aromatic C─H stretching (2850–2950 cm^−^^1^), C≡N (2240 cm^−^^1^), C═C aromatic bending (1610 cm^−^^1^), and C─O stretching (1000–1300 cm^−^^1^), in agreement with literature reports.^[^ ^37^, ^50^ ^]^ The spectra also showed characteristic peaks of [Emim][BF_4_], including C─H stretching of the imidazole ring (3150 cm^−^^1^), imidazole ring skeletal stretching vibrations (1570 and 1455 cm^−^^1^), and m‐substituted imidazole ring bands (845 and 765 cm^−^^1^).^[^ ^39^, ^51^ ^]^ Incorporation of [Emim][BF_4_] into the PIM‐1 layer resulted in the appearance and intensification of IL‐associated peaks, proportional to the IL content. The absence of new peaks in the spectra confirmed that no chemical reactions occurred, and the integration of [Emim][BF_4_] into PIM‐1 was purely physical. To evaluate the CO_2_ adsorption capacity of the selective layer, CO_2_ and N_2_ adsorption–desorption isotherms were measured for the PIM‐1/IL mixture at room temperature. N_2_ adsorption at 77 K revealed the high porosity of PIM‐1 and PIM‐1/20 wt% [Emim][BF_4_], with over 91% of pores <2–3 nm (Figures 2b and S6), suitable for CO_2_ adsorption given CO_2_’s kinetic diameter of 0.33 nm. Importantly, embodying 20 wt% [Emim][BF_4_] into PIM‐1 did not disrupt the microporous structure, as the isotherm showed a similar pattern to pure PIM‐1, albeit with reduced N_2_ uptake (Figure S6) and specific surface area (from 731 for PIM‐1 to 327 m^2^ g^−1^ for PIM‐1/20 wt% [Emim][BF_4_]) due to IL incorporation, but still retaining relatively high surface area and microporosity. For adsorption tests, the solution of PIM‐1 and IL in chloroform was freeze‐dried. The observed isotherms reflect the intrinsic microporosity of PIM‐1, acting as a micro‐reservoir for CO_2_. This permanent microporosity arises from inefficient polymer chain packing due to the spirocenter in the PIM‐1 backbone, and film processing does not compromise this structure. Higher CO_2_ uptake compared to N_2_ and O_2_ was observed, consistent with PIM‐1′s known CO_2_ affinity, supported by favorable CO_2_/N_2_/O_2_ permeation and adsorption selectivity reported in the literature.^[^ ^52^, ^53^ ^]^ Incorporation of [Emim][BF_4_] further enhanced CO_2_ uptake and CO_2_/N_2_/O_2_ selectivity (Figure 2c), owing to the IL's high CO_2_ solubility. Overall, PIM‐1′s microporous architecture serves as a scaffold for the CO_2_‐philic IL, significantly improving CO_2_ adsorption selectivity over N_2_ and O_2_.

The CO_2_ selectivity of the interlayer was evaluated by analyzing the mole fractions of CO_2_ and N_2_ in the permeate side of the CGDE under a low overpressure (1.1–1.2 psi) using a back‐pressure regulator and in‐line GC (Figure S7). This minor pressure difference closely mimics actual ECO_2_R conditions, which typically operate under ambient or slightly elevated pressures.^[^ ^54^ ^]^ Higher pressures would likely reduce selectivity, as the interlayer is partially permeable rather than fully gas‐tight, leading to non‐selective gas diffusion and diminished adsorption‐based selectivity of the CGDE. Notably, CO_2_, with a smaller kinetic diameter (3.3 Å) than N_2_ (3.64 Å) and O_2_ (3.46 Å), diffuses more readily through microporous media. Gas composition monitoring over time (Figure 2d) revealed an increase in the CO_2_ mole fraction from 15% in the feed to 65 ± 3% and 79 ± 4% in the permeate side of CGDEs with PIM‐1 and PIM‐1/20% [Emim][BF_4_], respectively. Correspondingly, N_2_ mole fraction decreased, resulting in CO_2_/N_2_ selectivity of 11.2 and 23.8 CGDE with pure PIM‐1 and PIM‐1/20% [Emim][BF_4_] layer, respectively. This selectivity stems from the low affinity of N_2_ for both PIM‐1 and [Emim][BF_4_], contrasted with CO_2_’s polar nature, which allows storage within the PIM‐1 micropores and physicochemical interaction with the ionic liquid. The established concentration gradient across the interlayer drives selective CO_2_ permeation and mass transport to the catalyst layer. Upon contact with the mixed gas, the interlayer preferentially adsorbs CO_2_, reducing the likelihood of N_2_ and O_2_ reaching the catalyst and thus increasing the local CO_2_ concentration. The gas permeation units (GPUs) of 2.1 × 10^3^ were calculated using a flow meter for the CGDE with PIM‐1/20 wt% [Emim][BF_4_] for CO_2_.

To gain engineering insights into gas transport through the interlayer and CO_2_ availability at the catalyst, COMSOL multiphysics simulations (details in Supporting Information) were conducted to examine the interlayer's CO_2_ purification from mixed gas feeds. As shown by the gas concentration profiles and contours (Figures 2e,f and S8), the PIM‐1/[Emim][BF_4_]‐based interlayer significantly reduced N_2_ and O_2_ partial pressures in the permeate, while CO_2_ partial pressure decreased only slightly. This outcome is expected given the IL‐based CO_2_‐philic microporous design, which ensures sufficient local CO_2_ availability near the catalyst and maintains ECO_2_R performance. The higher CO_2_ affinity of PIM‐1 and [Emim][BF_4_] (Figure 2c), combined with CO_2_’s smaller kinetic diameter (3.3 Å) compared to N_2_ (3.64 Å) and O_2_ (3.46 Å), facilitates preferential CO_2_ diffusion through the microporous PIM‐1 structure,^[^ ^55^ ^]^ resulting in markedly higher CO_2_ selectivity over N_2_/O_2_.^[^ ^56^, ^57^, ^58^ ^]^ A gas interaction index, representing the affinity between the selective layer and CO_2_, was also introduced into the model. The simulations showed that increasing this index, supposedly corresponding to higher [Emim][BF_4_] content in the interlayer, further sustained CO_2_ concentration within the layer (Figure S9), underscoring the importance of using CO_2_‐philic materials in GDE fabrication for mixed‐gas electrolysis. COMSOL Multiphysics simulations confirmed that at an interlayer thickness of ∼4–5 µm, the suppression of N_2_ and O_2_ concentration reached a plateau, indicating that the 5–7 µm layer used in this study is sufficient for selective transport with structural uniformity.

### Electrocatalytic Performance

**Figure 3:**
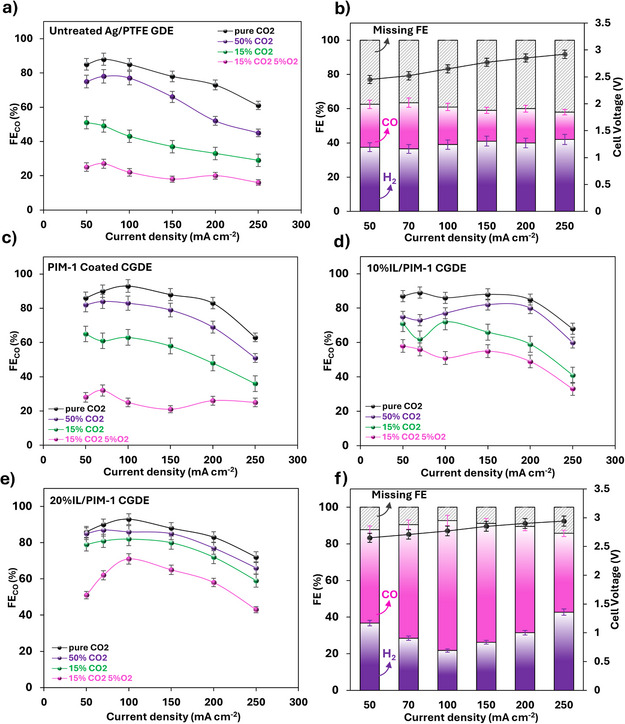
a) Faradaic efficiency (FE) of CO as a function of current density for pristine GDE with four gas feeds; b) FE of CO, H_2_, and missing FE for pristine GDE with 15% CO_2_, 5% O_2_ feed; c)–e) FE of CO as a function of current density for CGDEs with an interlayer in MEA electrolyzer using four gas feeds: pure CO_2_, 50% CO_2_ in N_2_, 15% CO_2_ in N_2_, and 15% CO_2_ with 5% O_2_ in N_2_; f) FE of CO, H_2_, and missing FE for CGDE with 20 wt% [Emim][BF_4_]/PIM‐1 interlayer with 15% CO_2_, 5% O_2_ feed. The current density‐FE curves were obtained with 30 min operation; error bars are from three repetitions.

Electrolysis tests were conducted in galvanostatic mode using a membrane electrode assembly (MEA) design, leveraging its scalability advantages for ECO_2_R, particularly for CO/syngas production.^[^ ^11^, ^59^, ^60^ ^]^ The setup employed a Ni foam anode, anion‐exchange membrane (PiperIon), and a CGDE cathode (Figure S10), with 1 M KOH as the anolyte. Electrochemical tests were performed at room temperature with Ag GDE and CGDEs containing varying [Emim][BF_4_] loadings, using four different gas feeds: pure CO_2_, 50% CO_2_ in N_2_, 15% CO_2_ in N_2_, and 15% CO_2_ with 5% O_2_ in N_2_ (simulated flue gas). Electrolysis tests were performed with humid gas feeds to better simulate post‐combustion flue gas. The CO_2_‐selective layer must exhibit hydrolytic stability, as exposure to humidity could lead to decomposition. PIM‐1 is inherently hydrophobic, water‐resistant, and stable, with no reports of water‐induced instability; it is also commonly used in water purification applications.^[^ ^61^, ^62^ ^]^ For the untreated GDE (without interlayer), CO Faradaic efficiency (FE) reached 80%–90% at current densities up to 150 mA cm^−^^2^ but decreased at higher currents as H_2_ generation increased (Figure 3a), consistent with reported Ag‐sputtered PTFE GDE performance.^[^ ^15^, ^63^ ^]^ Reducing CO_2_ concentration to 50% caused a moderate FE_CO_ drop and FE_H2_ increase, which became more pronounced with 15% CO_2_ in N_2_, reflecting CO_2_ dilution effects and limited availability at the catalyst. This trend was exacerbated by introducing 5% O_2_, where the combination of dilution and parasitic O_2_ reduction reaction (ORR) significantly compromised GDE performance. Unlike CO_2 _+ N_2_ feeds, where total FE_CO_ and FE_H2_ reached ∼95 ± 3%, the 5% O_2_ feed exhibited a substantial FE deficit around 40% (Figure 3b), attributed to ORR.

Incorporating the thin PIM‐1 layer slightly improved CO Faradaic efficiency (FE) under pure CO_2_ feed, achieving nearly 90% FE_CO_ at 150 mA cm^−^^2^ (Figure 3c). This enhancement stems from the microporous CO_2_‐adsorbent PIM‐1 layer, which locally stores CO_2_ beneath the catalyst, particularly crucial at higher current densities where CO_2_ availability limits performance.^[^ ^10^ ^]^ Unlike the untreated GDE, where CO_2_ delivery relies solely on gas flow diffusion, the PIM‐1 layer functions as a selective CO_2_ reservoir, owing to its high affinity for CO_2_ (Figure 2c). Increased local reactant availability near the catalyst boosts ECO_2_R performance, reducing H_2_ evolution caused by CO_2_ depletion.^[^ ^10^, ^34^, ^64^ ^]^ For the PIM‐1‐based CGDE, differences in FE_CO_ between pure CO_2_ and 50% CO_2_ feeds were less pronounced than for the untreated GDE, with performance gains more evident under 15% CO_2_ feed, achieving FE_CO_ > 60% at 100 mA cm^−^^2^ compared to 40% for the untreated sample. The PIM‐1 layer also promotes ECO_2_R by creating a triphasic system (solid polymer, water from the anode, and gas feed),^[^ ^65^, ^66^ ^]^ while its microporous structure modulates the reaction microenvironment, suppressing excessive bubble formation.^[^ ^47^ ^]^ The produced gases or diffused CO_2_ could be trapped near the catalyst layer due to the inhomogeneous gas delivery of the PTFE membrane with macroporous scaffold and rough surface, blocking the active sites, as witnessed in gas‐involving reactions.^[^ ^34^, ^45^, ^67^ ^]^ In contrast, the PIM‐1 layer fulfills a gas‐management role by regulating gas flux and improving the uniformity of CO_2_ delivery, enhancing the gas‐catalyst interface via triphasic microenvironments,^[^ ^68^, ^69^ ^]^ and selectively storing CO_2_.^[^ ^29^ ^]^ It should be noted that current collection in this system is achieved via the front side through the catalyst layer (Figure S10b), unlike in typical carbon‐based GDEs where current flows through the carbon substrate. As a result, incorporating the interlayer beneath the catalyst does not lead to a voltage increase (Figure S11). Online resistance measurements for GDEs with and without the PIM‐1/[Emim][BF_4_] interlayer also showed no significant difference (Figure S10c), confirming the effectiveness of this current collection strategy. Introducing 5% O_2_ further reduced FE_CO_ compared to O_2_‐free feeds, highlighting the PIM‐1 layer's limited O_2_ tolerance due to combined ORR parasitic effects, dilution by N_2_, and O_2_’s higher surface adsorption than N_2_. While performance remained superior to the untreated GDE, these results underscore the need to further enhance the interlayer's O_2_ tolerance.

To enhance CO_2_ uptake and catalytic performance, [Emim][BF_4_], a known CO_2_‐philic ionic liquid (IL) widely used in CO_2_ capture,^[^ ^39^ ^]^ was incorporated into the PIM‐1 matrix, forming a polymer‐IL composite layer. Beyond physical [Emim][BF_4_]‐CO_2_ interaction and CO_2_ trapping via its structure,^[^ ^70^, ^71^ ^]^ [Emim][BF_4_] exhibits (electro)chemical interactions with CO_2_ through its imidazole moieties,^[^ ^48^ ^]^ and its catalytic activity, particularly for CO_2_‐to‐CO conversion, has been reported.^[^ ^19^, ^48^, ^72^ ^]^ Unlike conventional approaches where [Emim][BF_4_] is used as an electrolyte or additive,^[^ ^73^ ^]^ we examined its effect when integrated directly into the GDE, adjacent to the catalyst layer. The electrocatalytic performance of CGDE tests demonstrated that IL incorporation expanded the current density range for achieving FE_CO_>90% under pure CO_2_ feed and suppressed H_2_ evolution (Figure 3d,e). This improvement arises from two key factors: (1) enhanced CO_2_ capture within the composite layer, aligning supply with catalytic consumption during ECO_2_R; and (2) a co‐catalytic effect at the interface between the IL composite layer and the silver catalyst. [Emim][BF_4_] attracts CO_2_ owing to its fluorinated anion, and due to its rather small molar volume as compared with other ILs, it has a smaller Herny's constant and consequently a higher gas solubility or capture CO_2_ uptake capacity (based on regular solution theory $\ln H=\alpha +\frac{\beta }{V_{IL}^{4/3}}$, a smaller IL molar volume, *V*_IL_, results in a smaller Henry's constant (H) thus a higher gas solubility).^[^ ^39^, ^74^ ^]^ Combined with PIM‐1, this yields a microporous IL‐based layer with elevated CO_2_ uptake^[^ ^75^ ^]^ and a modulated reaction microenvironment, promoting triple‐phase interface formation between the interlayer and Ag layer.

In the feed containing 5% O_2_, incorporation of [Emim][BF_4_] into the PIM‐1 layer improved performance compared to the pure PIM‐1 CGDE. Unlike N_2_, which is inert, O_2_ reduction competes with ECO_2_R, requiring a thermodynamically 1.0 V lower potential.^[^ ^76^, ^77^, ^78^ ^]^ While impurities like NO*_X_* and SO*_X_* can be removed by established alkaline absorbers,^[^ ^79^ ^]^ O_2_ removal at low concentrations remains costly, making O_2_ tolerance in GDEs critical.^[^ ^80^ ^]^ O_2_ reduction typically occurs on both the conductive gas‐diffusion layer (GDL) and catalyst.^[^ ^81^ ^]^ However, FE_CO_ comparisons between feeds with and without O_2_ (Figure 3d,e) revealed smaller changes after [Emim][BF_4_] addition, attributed to the synergistic effects of [Emim][BF_4_] reinforcing O_2_ tolerance via suppressed O_2_ adsorption on PIM‐1 (Figure 2c) and favorable CO_2_‐IL interactions.^[^ ^82^, ^83^, ^84^ ^]^ This was corroborated by significantly reduced missing FE (attributed to O_2_ reduction) as shown in Figure 3f. Additionally, the non‐conductive substrate used here contrasts with conventional carbon‐based GDLs that promote parasitic O_2_ reduction.^[^ ^81^, ^85^ ^]^ Thus, by employing a non‐conductive substrate and an optimized CO_2_‐selective layer beneath the catalyst, the adverse effects of O_2_ are effectively mitigated.

**Figure 4:**
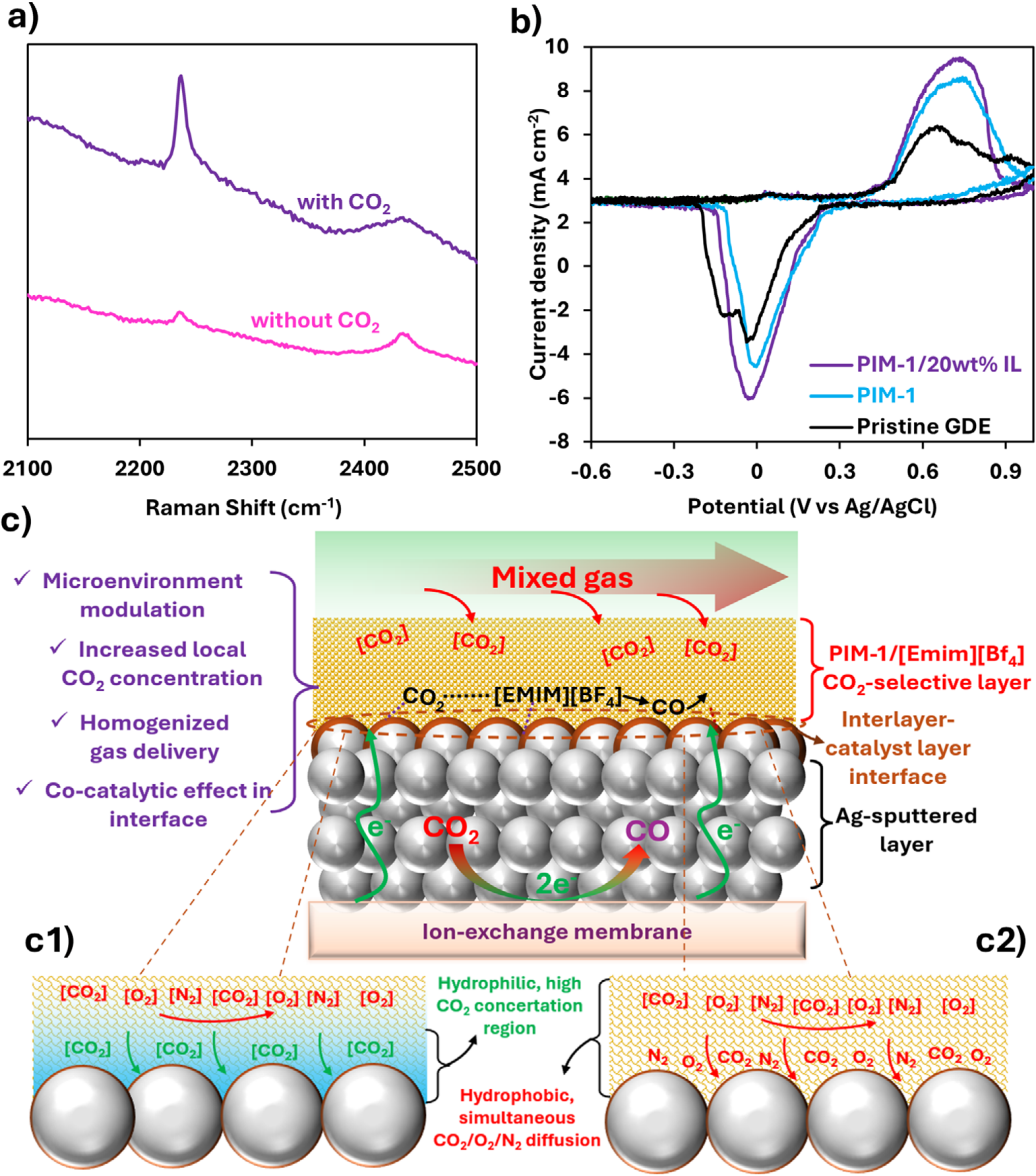
a) Raman spectra of [Emim][BF_4_] under CO_2_‐free and CO_2_‐saturated conditions, showing stronger peaks in the presence of CO_2_; b) Cyclic voltammetry of hydroquinone solution at 60 mV s^−1^ for various GDEs; larger peaks for composite GDEs indicate increased electroactive surface area; c) Schematic illustrating the interaction between CO_2_ and the interlayer, highlighting its multifunctional roles in microenvironment modulation, enhanced CO_2_ availability, uniform delivery, and co‐catalytic effects (interaction between [Emim][BF_4_] in the CO_2_‐selective interlayer and CO_2_ could be through formation of complexes and intermediates (e.g., CO_2_‐[Emim], [EMIM–COOH]^−^) to promote CO_2_‐to‐CO). Two scenarios for the interlayer, with (c1) and without [Emim][BF_4_] (c2), are shown to depict the effect of local hydrophilicity to suppress O_2_ diffusion as compared with CO_2_.

To get more insights on the interaction of [Emim][BF_4_] with CO_2_ and its role in enhancing the catalytic activity of the CGDE, Raman spectra of [Emim][BF_4_] and PIM‐1 under CO_2_‐free and CO_2_‐saturated conditions at ambient pressure were collected, confirming visible interactions with CO_2_ (Figures 4a and S12). Specifically, peaks at 1280 and 1385 cm^−1^ in [Emim][BF_4_] correspond to the Fermi resonance of CO_2_, while increased intensity at 2238 and 1000–1600 cm^−1^ confirms PIM‐1–CO_2_ interactions via C─C and C─O functional groups.^[^ ^86^, ^87^, ^88^ ^]^ Prior studies have also demonstrated strong CO_2_ interactions with the imidazolium ring and alkyl tail of [Emim][BF_4_].^[^ ^88^, ^89^ ^]^ There have been studies detailing the catalytic interface modulation of PIM‐1,^[^ ^34^ ^]^ and electrocatalytic effect of [Emim][Bf_4_] for ECO_2_R, specifically CO_2_‐to‐CO.^[^ ^90^ ^]^ [Emim][BF_4_] modulates the reaction microenvironment through synergistic participation of both its cations and anions,^[^ ^73^, ^91^ ^]^ by hydrolysis of [BF_4_]^−^ in the presence of water/moisture, leading to a greater proton availability and improving ECO_2_R‐to‐CO.^[^ ^90^, ^92^ ^]^ CO_2_‐[Emim] complex formation via [Emim] interaction with CO_2_ can be converted into adsorbed CO, lowering the energy barrier for CO_2_‐to‐CO conversion and suppressing H_2_ evolution.^[^ ^19^, ^93^ ^]^ Additionally, the imidazole cation could chemically interact with CO_2_ and [Emim]^+^ acts as a proton donor,^[^ ^94^ ^]^ promoting the proton‐coupled electron transfer,^[^ ^95^, ^96^, ^97^ ^]^ resulting in a lower energy barrier for the formation of the CO_2_*^−^ radical anion,^[^ ^98^ ^]^ similar to the co‐catalytic effect observed for pyridine‐containing materials near the catalyst in ECO_2_R.^[^ ^10^, ^99^, ^100^ ^]^ [Emim]^+^ also induces electric field effects in the interface of the electrode and imidazolium (catalyst layer and interlayer interface), facilitating the first electron transfer in the ECO_2_R,^[^ ^19^, ^40^, ^41^ ^]^ similar to polymerized imidazole‐based IL coatings.^[^ ^101^ ^]^ Mechanistic studies confirm that [Emim][BF_4_] not only mediates CO_2_‐to‐CO by stabilizing intermediates but also acts as an electrocatalyst, forming an [EMIM–COOH]^−^ intermediate that decomposes to CO under applied potential.^[^ ^48^, ^95^ ^]^ These studies provide compelling evidence for the catalytic, mediating, and microenvironment‐modulating roles of [Emim][BF_4_] during ECO_2_R.^[^ ^102^ ^]^ Moreover, the formation of CO_2_‐IL complexes observed in these works supports the present study, where the IL is integrated within the CGDE. The composite layer, humidified by feed gas and water transport from the anode, enhances [Emim][BF_4_]’s intrinsic proton conductivity as active sites.

The contribution of the selective layer to the electrochemically active area and CO_2_ storage capacity was evaluated using cyclic voltammetry (CV) with 2 mM hydroquinone as a redox probe in 0.2 M KOH.^[^ ^67^ ^]^ At 60 mV s^−^^1^, the peak current corresponding to hydroquinone redox reactions increased for GDEs with the selective layer (Figure 4b), with a more pronounced enhancement for CGDEs incorporating [Emim][BF_4_]. The IL imparts ionic conductivity, while PIM‐1 functions as a microporous scaffold that confines ILs, forming a quasi‐solid electrolyte beneath the catalyst.^[^ ^103^ ^]^ According to the Randles‐Ševčík relationship (*I*_p_ = (2.69 × 105) × *n* ^3/2^AD^1/2^Cν^1/2^), the peak current (*I*_p_) is directly proportional to the electroactive surface area (A) of the electrode under diffusion‐controlled conditions.^[^ ^104^ ^]^ The observed shift could be attributed to the existence of polymer‐Ag boundary and the presence of IL in the microporous layer, making the complex interface for the redox cycle.^[^ ^105^ ^]^ The increased redox response indicates improved accessibility of hydroquinone to active sites, consistent with previous studies showing polymer‐IL composite layers enhancing ion transport, electroactive surface area (Figure S13), and charge transfer.^[^ ^45^, ^68^ ^]^ PIM‐1′s intrinsic microporosity, combined with [Emim][BF_4_]’s ionic conductivity, facilitates enhanced molecular diffusion and redox activity.^[^ ^106^, ^107^ ^]^ Therefore, the observed enhancement in hydroquinone redox peaks provides evidence for electrocatalytic contribution of PIM‐1/[Emim][BF_4_], making it justifiable to say that the selective layer could interact with CO_2_ and its interface with the silver layer acts as catalytic active environment due to the presence of moisture, proton, and high CO_2_ local concentration.^[^ ^108^ ^]^ The schematic in Figure 4c illustrates the multifunctional role of the PIM‐1/[Emim][BF_4_] interlayer, including CO_2_ capture, microenvironment modulation, and possible electrocatalytic contributions at the interface with the silver catalyst. While direct operando observation is beyond our focus and is not easily feasible due to the CGDE design and MEA configuration, ex situ Raman data, electrochemical probing (Figure 4b), and literature precedent support the mechanism that the interlayer participates in ECO_2_R. The broadened current density range achieving FE_CO_ > 80% for CGDEs with [Emim][BF_4_] corroborates the conclusion for the composite layer's contribution to ECO_2_R.

The O_2_ tolerance of the designed CGDE can be further explained by the water management properties of the interlayer, which modulate the rate‐determining step of ORR^[^ ^109^ ^]^ and adjust local pH to favor ECO_2_R over ORR.^[^ ^110^ ^]^ This stems from the interlayer's lower hydrophobicity compared to the PTFE substrate and its microporous architecture, enabling modest water uptake. The hydrophilicity of the electrode and local H_2_O availability critically influence MEA‐based ECO_2_R performance.^[^ ^111^ ^]^ Coating PTFE with PIM‐1 slightly reduced the contact angle from 144° to 139° (Figure S14), while incorporating 20 wt% [Emim][BF_4_] further decreased it to 108°, reflecting the IL's inherent hydrophilicity.^[^ ^46^ ^]^ O_2_, being volatile in aqueous solutions, exhibits high mass flux in hydrophobic networks (7.7 × 10^−^^3^ mol m^−^^2^ s^−^^1^, Wilke‐Chang correlation) but much lower flux in hydrophilic networks (4.4 × 10^−^⁴ mol m^−^^2^ s^−^^1^, Bosanquet relation).^[^ ^112^, ^113^ ^]^ In contrast, CO_2_, with lower volatility, shows comparable transport rates in hydrophilic and hydrophobic networks (2.7 × 10^−^^2^ versus 3.6 × 10^−^^2^ mol m^−^^2^ s^−^^1^). Thus, leveraging the differences in gas volatility between CO_2_ and O_2_, a more hydrophilic interlayer selectively imposes mass transport resistance against O_2_, enhancing the O_2_ tolerance of the CGDE,^[^ ^114^ ^]^ as described in Figure 4c1,c2.

A brief cost‐estimation benefit was conducted to assess the feasibility of integrating CO_2_ capture and ECO_2_R within a single CGDE‐based system under certain conditions.^[^ ^115^ ^]^ The estimation assumes that the CGDE effectively eliminates separate CO_2_ capture, compression, and transport steps (as illustrated in Figure 1a), while in the conventional segregated design, CO_2_ capture and ECO_2_R occur at geographically separated sites, necessitating compression and transport. For the segregated scenario, costs associated with CO_2_ capture, transport, and compression were included in calculating the minimum selling price of CO (MSP_CO_). In contrast, these costs were excluded from the integrated design, though a slightly higher electrolyzer capital cost was considered due to the inclusion of capture materials and advanced design features. The PIM‐1 coating on the PTFE substrate can be achieved via kiss‐coating, a standard technique in electrode/membrane fabrication, so only the cost of PIM‐1 material was added to the integrated design. MSP_CO_ calculations (details in Supporting Information) compared integrated and segregated scenarios and also estimated potential costs under ideal, optimized conditions. CO_2_ capture costs depend on feed concentration, ranging from $30 to $300 per ton of CO_2_, with an average of $180 per ton of CO_2_ assumed here.^[^ ^116^, ^117^ ^]^ CO_2_ transport‐compression costs, including pipeline construction (length, diameter), compression/booster stations, materials, labor, right‐of‐way, and operational costs, were estimated at $230 per ton CO_2_ for a 1000 km pipeline.^[^ ^27^, ^28^, ^118^, ^119^, ^120^ ^]^ For product separation, pressure swing adsorption (PSA) was considered, as it is commercially feasible for separating high‐purity CO from N_2_, H_2_, O_2_, and excess CO_2_. While PSA is efficient for CO separation in the segregated scenario, the presence of N_2_ and O_2_ in the integrated design increases complexity and cost.^[^ ^121^, ^122^ ^]^

**Figure 5:**
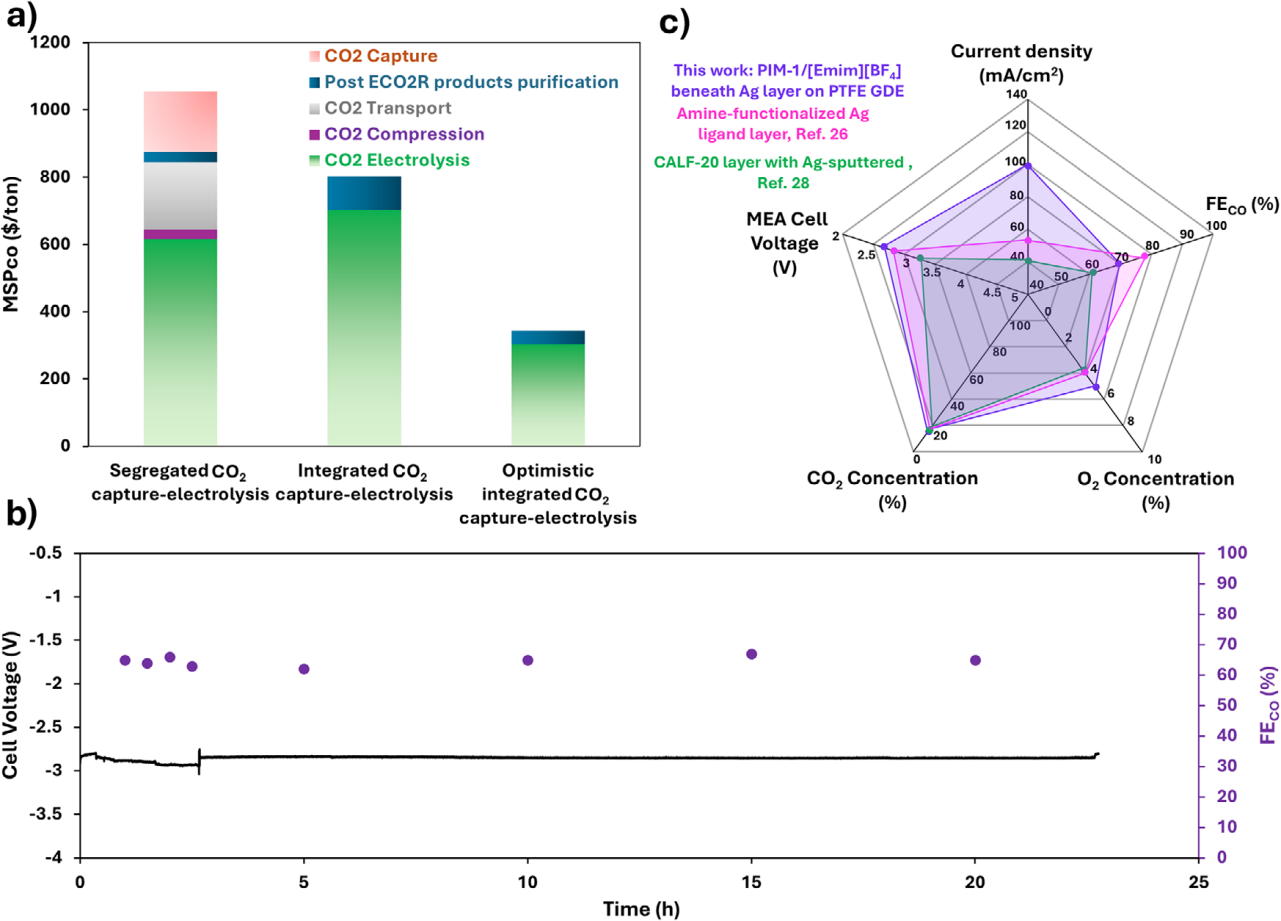
a) Breakdown of cost contributors to the minimum selling price of CO (MSP_CO_) for integrated and separated systems (parameters in SI), with an additional scenario modeled at 200 mA cm^−^^2^, 1.6 V, and 95% FE; b) Stability test of MEA electrolyzer with CGDE containing PIM‐1/20% [Emim][BF_4_] interlayer, operated at 150 mA cm^−^^2^; c) Spider diagram comparing key metrics of this work with other MEA‐based CO production studies using O_2_‐containing CO_2_ feeds.

Comparison of the cost results for conventional and integrated scenarios revealed that the integrated design could reduce the MSP_CO_ by around 25% compared to the conventional route (Figure 5a), based on performance metrics of 80% FE_CO_ at 200 mA cm^−^^2^ for the separated scenario (pure CO_2_ feed) and 65% FE_CO_ at 150 mA cm^−^^2^ (2.82 V) for simulated flue gas feeding. Although the integrated MSP_CO_ remains higher than current industrial standards, this brief cost estimation, primarily comparing integration versus separation, exhibits consistency with comprehensive ECO_2_R technoeconomic analysis in the literature,^[^ ^123^ ^]^ excluding common costs such as CO_2_ price. Notably, targeting syngas instead of CO as the product could further enhance economic viability, given its compatibility with established downstream processes and the ability to operate at higher current densities. Considering an optimistic scenario with advanced electrolyzer performance (95% FE, 1.6 V cell voltage) could halve the MSP_CO_ compared to current experimental metrics, underscoring the significant impact of improvements in electrocatalyst, electrode, and electrolyzer design. This analysis highlights the potential advantages of integrated designs, though outcomes will depend on variables such as renewable electricity availability, CO_2_ feed concentration, and the distance between capture and conversion sites, which influence transport and compression costs.

To evaluate long‐term, the CGDE‐containing 20 wt% [Emim][BF_4_] was measured at 150 mA cm^−2^ with a mixed feed (15% CO_2_, 5% O_2_, N_2_ balance). Figure 5b shows that stable operation over 20 h can be achieved while maintaining an FE_CO ∼_ 65%. The lack of degradation highlights the notable stability performance of our CGDEs. Under these conditions, a CO/H_2_ ratio of ∼2.5 was achieved, demonstrating effective direct syngas production from simulated flue gas. SEM images of the CGDE post‐operation showed no significant structural change (Figure S15) attributable to instability of the PIM‐[Emim][BF_4_] layer or leaching of [Emim][BF_4_] from the PIM‐1 matrix. While prior studies have reported ionomer loss from the catalyst layer in flowing catholyte electrolyzers,^[^ ^124^ ^]^ the current MEA design uses [Emim][BF_4_] in a solid‐state form embedded within the PIM‐1 scaffold. This configuration minimizes leaching risks. Additionally, although [Emim][BF_4_] has been reported to degrade under highly cathodic conditions (>3.6 V),^[^ ^41^, ^125^ ^]^ the lower operating voltages used here and the solid‐state environment ensured stable performance. ICP‐MS analysis of the anolyte and AEM (after acid digestion, in case of retained [BF_4_]^−^) showed no detectable boron accumulation, ruling out [BF_4_]^−^ migration and confirming the chemical stability of the CGDE. A comparison with literature shows that our design outperformed previous studies on gas‐phase CO_2_‐to‐CO reduction from impure CO_2_ streams in terms of CO partial current density (Table S1), particularly for MEA electrolyzers operating with flue gas‐like concentrations (up to 15% CO_2_ in O_2_‐containing feeds) (Figure 5c). This highlights the potential of the approach and underscores the importance of thoughtful GDE design, from substrate selection to precise tuning of the thin selective layer.

## Conclusion

This study presents a strategy for integrating solid‐state CO_2_ capture with electrochemical CO_2_ reduction (ECO_2_R) within the gas‐diffusion electrode (GDE) structure. By carefully selecting CO_2_‐philic materials and engineering a CO_2_‐selective composite GDE, we enabled efficient delivery of CO_2_ from mixed feeds with as low as 15% CO_2_ and 5% O_2_. The complementary roles of the PIM‐1 matrix and CO_2_‐selective [Emim][BF_4_] created a thin, porous, micro‐reservoir layer beneath the catalyst, ensuring localized CO_2_ availability. The electrochemical activity of [Emim][BF_4_], its interaction with CO_2_, and the microporous structure of PIM‐1 collectively enhanced ECO_2_R performance. Notably, the CGDEs achieved FE_CO_>70% at 100 mA cm^−^^2^ using simulated flue gas. Multiphysics simulations confirmed the critical role of CO_2_ affinity in maintaining CO_2_ concentration within the interlayer and suppressing O_2_/N_2_ permeation to the catalyst. The cost estimation benefit analysis indicated that the integrated design reduced the minimum selling price of CO by over 25% compared to the conventional segregated capture‐electrolysis route, with potential for >50% cost reduction under optimal operating conditions. Future work will focus on functionalizing PIM‐1 with redox‐active groups (e.g., pyridines) to enhance electron mediation in MEA‐based ECO_2_R, investigating microenvironment modulation via operando techniques, incorporating metal nanoparticles (e.g., Cu or Ag) into PIM‐1 to boost conductivity and catalytic activity, and considering other ionic liquids with different anion/cation to investigate their effect to regulate ECO_2_R with less pure CO_2_ feed, particularly with SO*_x_*/NO*_x_* impurities. Additionally, targeting liquid products may offer a more economical path by simplifying post‐electrolysis purification in mixed gas scenarios.

## Conflict of Interests

The authors declare no conflict of interest.

## Supporting information



Supporting information

## Data Availability

The data that support the findings of this study are available from the corresponding author upon reasonable request.
