# Supplementary material for: From Flue Gas to Syngas: Composite Electrode Based on Ionic Liquid and Microporous Polymer for MEA‐Based CO2 Electrolysis
Source: Angew Chem Int Ed Engl. 2025 Aug 14;64(40):e202513103. doi: 10.1002/anie.202513103 (PMC12462752; doi:10.1002/anie.202513103)
Supplement: Supplementary file 1 — Supporting information [file ANIE-64-e202513103-s001.pdf]

Supporting Information

From Flue gas to Syngas: Composite Electrode based on Ionic Liquid and Microporous Polymer for MEA-based CO_2 Electrolysis

Hesamoddin Rabiee [a*], Abhijit Dutta [a,b], Penghui Yan [c], Lei Ge [d], Fatereh Dorosti [c,d], Xin Yu [a,b], Alain Rieder [a,b], Peter Broekmann [a,b]

[a] Dr Hesamoddin Rabiee, Dr Abhijit Dutta, Xin Yu, Alain Rieder, Prof. Peter Broekmann
Department of Chemistry, Biochemistry and Pharmaceutical Sciences, University of Bern, Freiestrasse 3, Bern 3012, Switzerland. E-mail: hesamoddin.rabiee@unibe.ch

[b] Dr Abhijit Dutta, Xin Yu, Alain Rieder, Prof. Peter Broekmann
NCCR Catalysis, Switzerland

[c] Dr Penghui Yan, Dr Fatereh Dorosti
School of Chemical Engineering, The University of Queensland, Brisbane, QLD 4072, Australia

[d] Prof. Lei Ge, Dr Fatereh Dorosti
Centre for Future Materials, University of Southern Queensland, Springfield, QLD 4300, Australia

Experimental Section

Fabrication of composite GDEs. The composite GDEs (CGDEs) with a CO_2-selective layer were fabricated by adding a layer of PIM-1 on the PTFE GDL (Oleophobic, Polypropylene Backer, 0.45 Micron pore size, Tisch Scientific) before catalyst loading. 2wt% PIM-1 solution (0.2 g in 9.8 g of chloroform) was used to coat the PTFE GDL, and since the solution had a very low viscosity it led to a thin layer. A piece of PTFE GDL was taped on a stainless-steel plate, and the tape covered the surrounding completely. Subsequently, the PTFE was kept in 45° angle and the PIM-1 solution was poured on the GDE and was let to dry. This procedure was repeated until the desired thickness was achieved. This method of coating imitates "kiss-coating" technique which is common in fabrication of thin film membranes [1, 2]. Increasing the number of repetitions results in a thicker later of PIM-1 on the PTFE. For the case of PIM-1/[Emim[BF_4] coating, [Emim][BF_4] was added to the solution in different weight ratios relative to PIM-1 from 0 to 20wt%. The coated GDL was let dry in oven at 60 °C to remove solvent residues. Afterwards, a layer of silver was sputtered on the selective layer with a thickness of 400 nm [3].

Electrochemical Reduction of CO_2. ECO_2R experiments were conducted in a membrane-electrode assembly type electrolyzer (Dioxide Materials). A mass flow controller was used to adjust the CO_2 (99.9%, Carbagas, Switzerland) flow rate (30 ml min^{-1}) (Voegtlin, Switzerland, ±1% resolution). The electrochemical measurement was done by an Autolab (Metrohm) potentiostat. The GDEs were used as the cathode (working electrode) with Ni foam as the anode, and they were separated by an anion exchange membrane (PiperIon). 1.0 M KOH was use as the anolyte. Choosing alkaline anolyte let us to use a non-noble anode. The feed gas was humidified by passing it through a humidifier. GDE area of 1 cm × 1 cm was exposed to the electrolyte as the active area for electrolysis. A slight gas feed pressure of 1.1-1.2 psi was applied to mimic the conditions used for the breakthrough test.

A gas chromatograph (GC 8610C, SRI Instruments) equipped with a thermal conductivity detector (TCD) and a flame ionization detector (FID) was connected to gas outlet of the electrolyzer to analyze the composition of gaseous products. Hydrogen (H_2, 99.999%) and argon (Ar, 99.999%) were used as the carrier gases for the FID and the TCD, respectively. Air was used as the balance gas for the FID. The FE of gaseous products was determined via $FE_i = \frac{e_i \times F \times P \times V \times X_i}{J \times R \times T} \times 100$, where e_i is the electron transfer required (in mole) to generate one mole of a gas product, X_i represents the products concentration in the reactor gas outlet measured with the mass spectrometer, V is the outlet gas volumetric flow rate (ml min^{-1}), P is the atmospheric pressure (101.3 kPa), and J is the current (mA) (from the potentiostat).

Characterizations

Zeiss DSM 982 was used to achieve scanning microscopy (SEM) images. CO_2 adsorption–desorption isotherm was obtained by TriStar Micromeritics. The surface morphology of the GDEs was analyzed by means of white light interferometry (Contour GT, Bruker) with 50x magnification lens. Vision64 software (Bruker) was used for operating the instrument and the data analysis. Raman spectroscopic analyses were conducted using a LabRAM HR800 confocal microscope (Horiba Jobin Yvon). Spectral data were collected with the Raman spectrometer coupled to a confocal microscope (Horiba Jobin Yvon) and operated by the Lab Space 3.0 software. The calibration was carried out using a silicon wafer standard (520.6 cm^{-1}). A large working distance objective lens (50 × magnification, 8 mm focal length) has been applied with a numerical aperture of 0.1 in order to focus a diode-pumped solid-state (DPSS) laser beam (excitation wavelength of 532 nm; laser power of 3 mW for standard Raman measurements) on the sample and to collect both the incident and scattered laser light. FIB-SEM was done by the Thermo Scientific Scios 2 DualBeam machine. A Focussed Ion Beam scanning electron microscope SCIOS 2 (Thermofischer, Waltham, MA, USA) was used to create cross sections. Practically, a 500 nm layer of Pt was deposited using the gas injection system to protect the underlaying structure. Then, a cross section was milled with a Ga^{2+} ion beam at a stage tilt of 52° using a current of 1 nA. The cross sections surface was polished with an ion beam with a beam current of not higher than 100 pA. Finally, electron micrographs were recorded using in-beam Everhart–Thornley secondary electron detector and the Trinity (T1) in-beam back scatter electron detector with a dwell time of no less than 10 microseconds. Fourier transform infrared (FTIR) spectra of the electrodes were achieved using a Perkin Elmer Frontier FTIR (600-4000 cm^{-1}). Ag sputtering target (AJA International, 99.995%) was sputtered at 100 W in direct current mode, with 3 mTorr Ar plasma for 10 minutes (AJA International Orion series), yielding a nominal thickness of ~400 nm. The pressure-dependent permeability test was done using Porolux 1000 using N_2 gas. ICP Mass Spectrometery analysis was done using a NexION 2000 ICP-MS (PerkinElmer) with Kinetic Energy Discrimination (KED) mode (helium as the collision gas), calibrated with multielement standard with 10 ppb detection sensitivity. To investigate migration of $[BF_4]^-$ to the anode side through AEM, samples of anolyte before and after long-term test, and acid-digested AEM (after digestion in concentrated HNO_3 for 3 h at 60 °C in case of retained $[BF_4]^-$ in AEM structure) were tested for boron (B) by ICP-MS. Comparing the boron values in anolyte before and after the electrolysis indicates any boron accumulation in the anolyte.

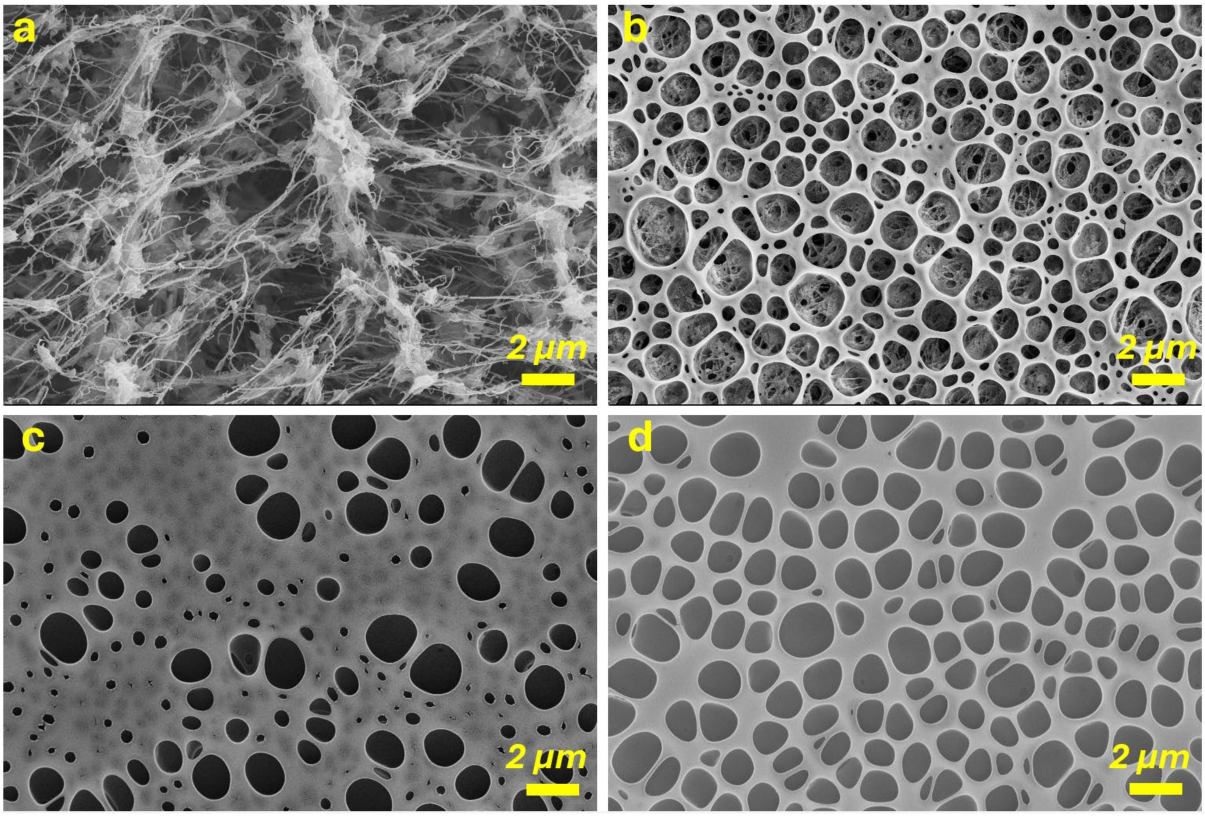

Fig. S1. The surface SEM image of a) PTFE substrate; and PTFE coated by PIM-1 via layer-by-layer coating for b) 5, c) 10, and d) 15 cycles. After 15 cycles the PTFE substrate is fully covered, and its surface cannot be seen. The holes on the surface are due to solvent evaporation. After 15 cycles coating, layers come on top of each other and therefore these holes are be pinholes.

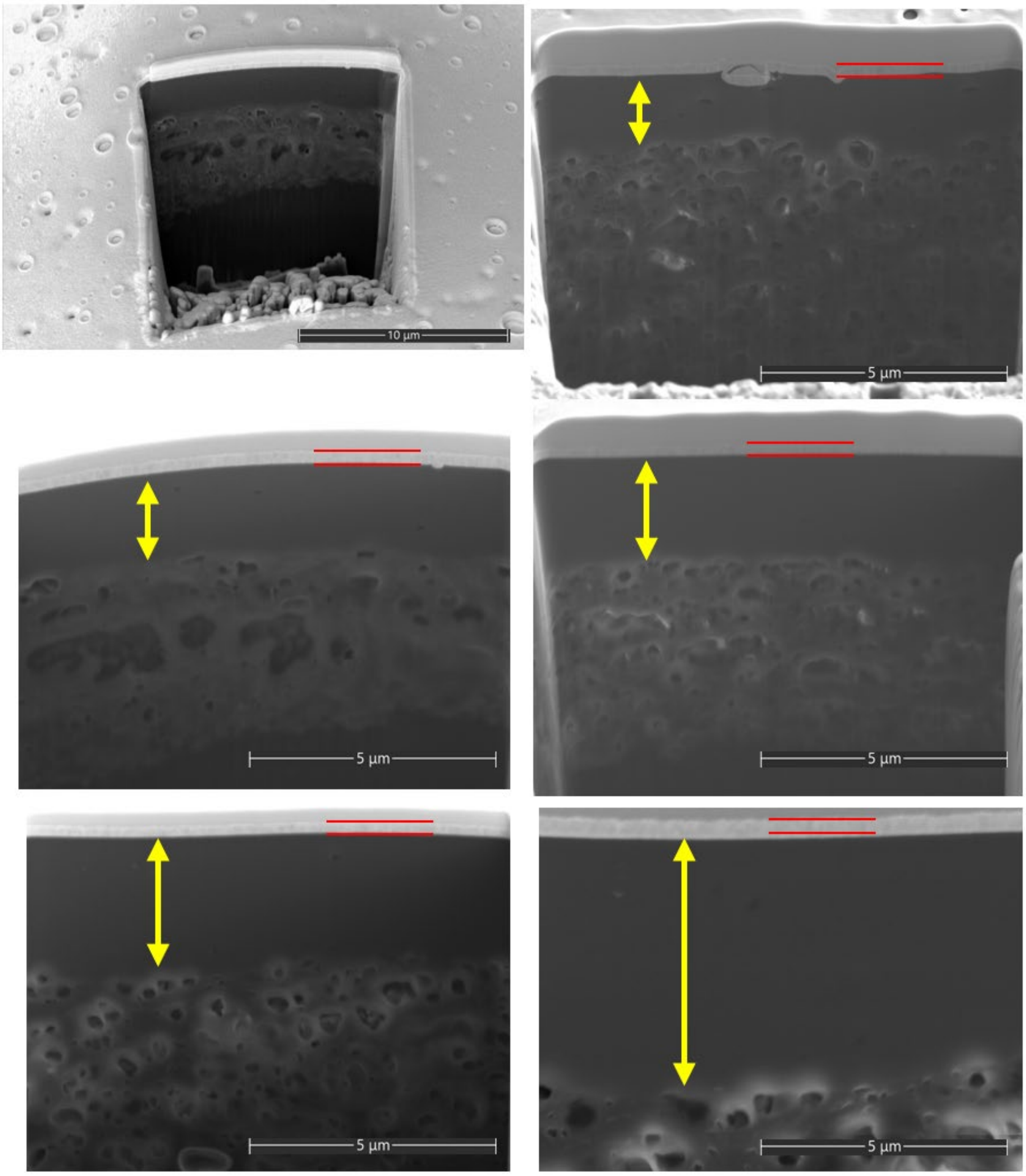

Fig. S2. FIB-SEM cross-sectional images of the CGDEs with different interlayer thickness because of increasing the coating cycles (1, 3, 5, 10, 15 cycles).

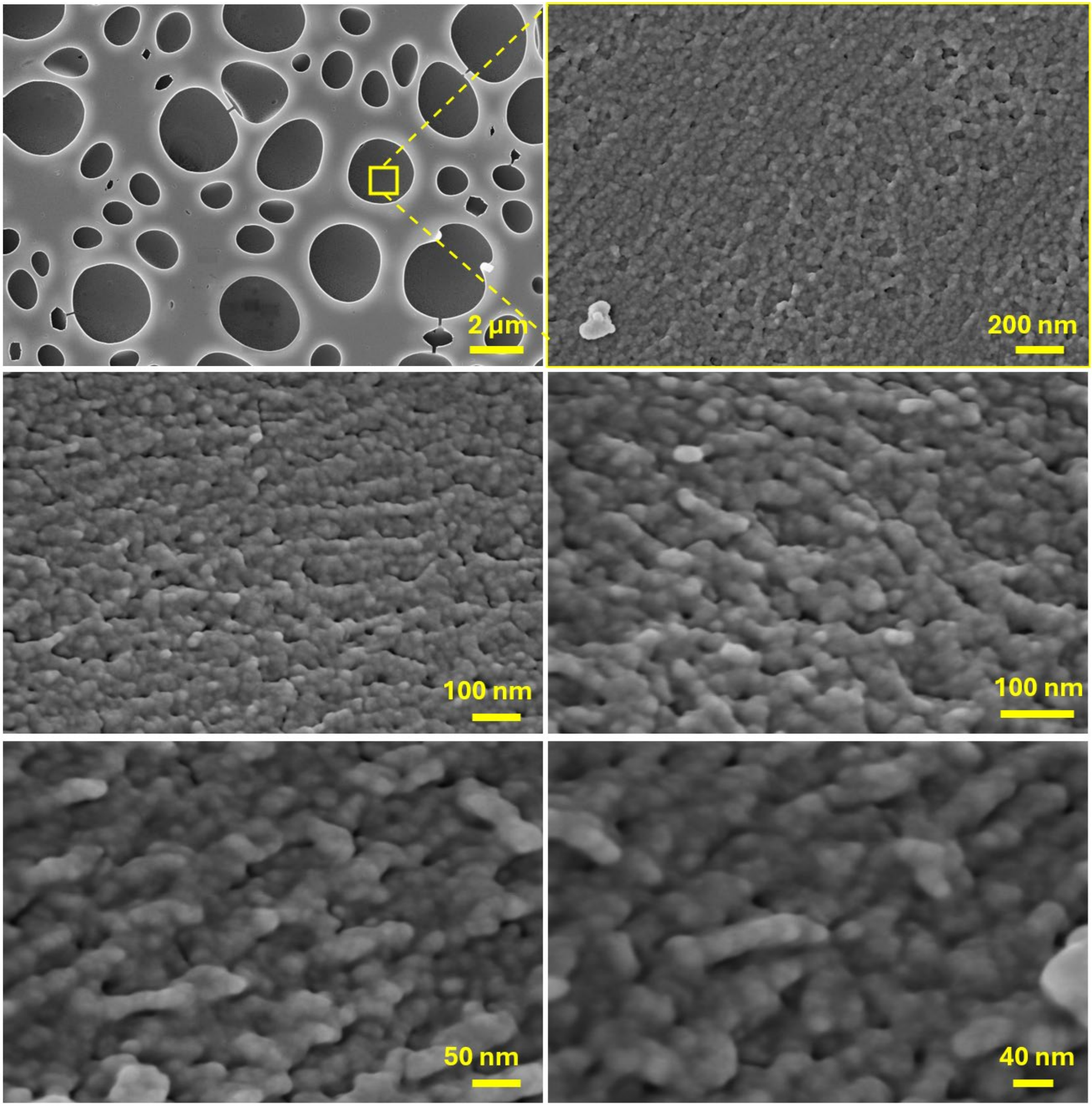

Fig. S3. High-magnification SEM images of the PIM-1/20wt% [Emim][BF_4] layer, showing the micropores which sieve the gas and do not fully block the permeation.

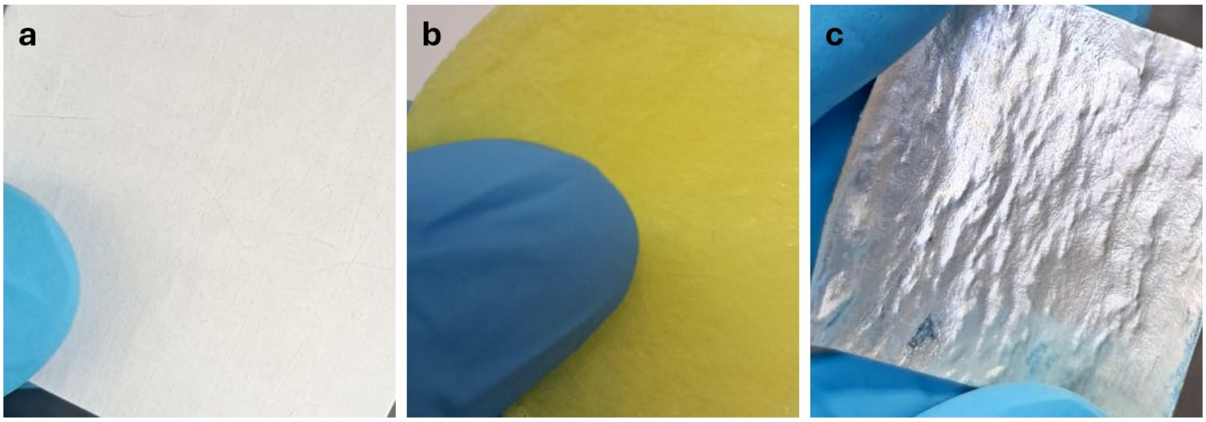

Fig. S4. The image of a) PTFE substrate, b) PTFE Substrate coated by PIM-1/[Emim][BF_4] layer, c) Ag layer sputtered on the PIM-1/[Emim][BF_4] layer.

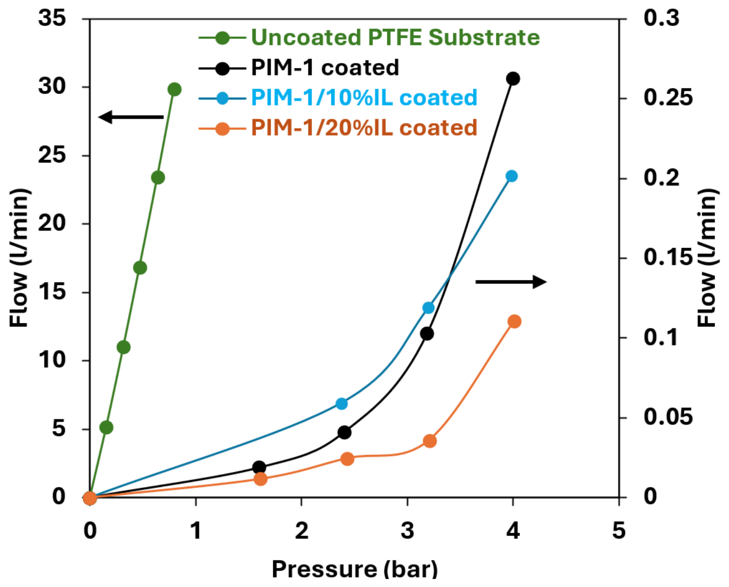

Fig. S5. The flux of N_2 into the different GDEs at elevated pressures. For the PTFE substrate a very high flow was observed in low pressures, indicating the inhomogeneous structure for gas delivery.

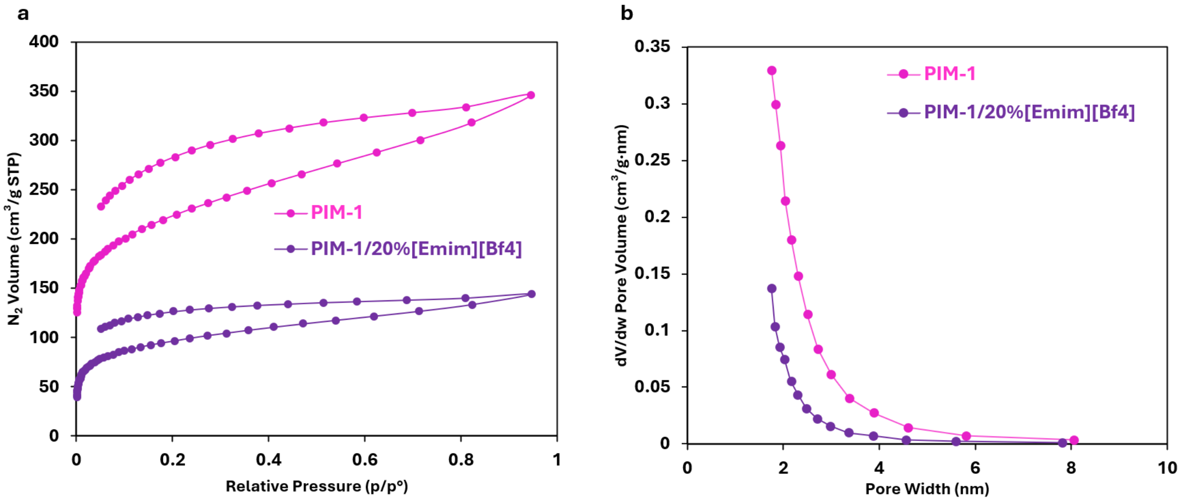

Fig. S6. a) N_2 sorption isotherm collected at 77 K for and **b)** pore size distribution of PIM-1 and PIM-1/20wt%[Emim][BF_4]. In corporation of [Emim][BF_4] into PIM-1 led to decrease in specific surface area and consequently N_2 uptake, however still retaining high specific area and microporous structure.

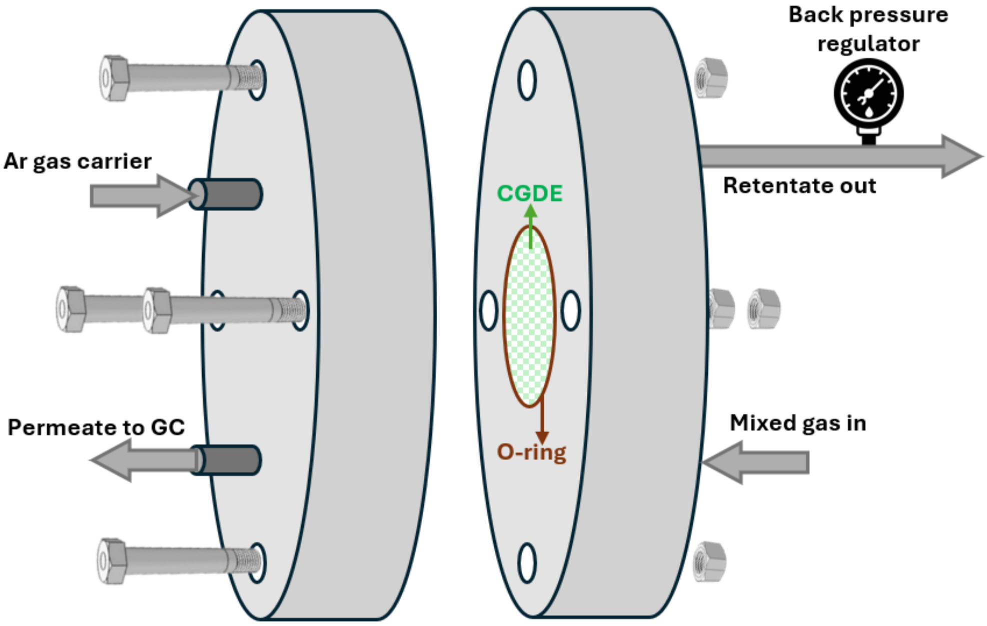

Fig. S7. The cell to measure the permeation of mixed gas through the CGDE. Mixed gas is fed from right side, and its pressure is adjusted (1.1-1.2 psi) using a back pressure regulator. On the left side the permeate gas is carried by Ar to GC to measure the concentration of gasses.

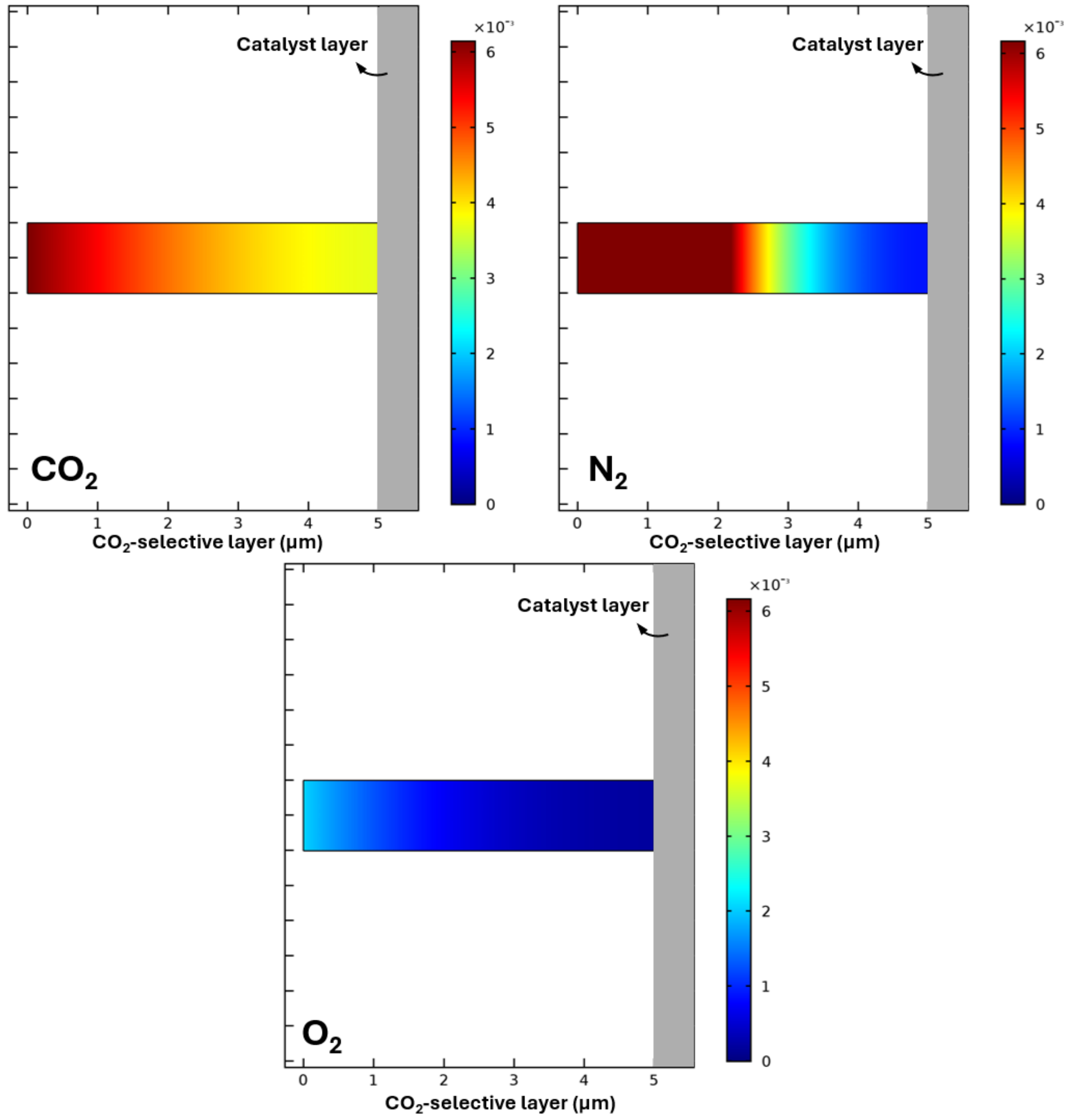

Fig. S8. COMSOL simulation of concentration contours of CO_2, N_2 and O_2 in the CO_2-selective layer.

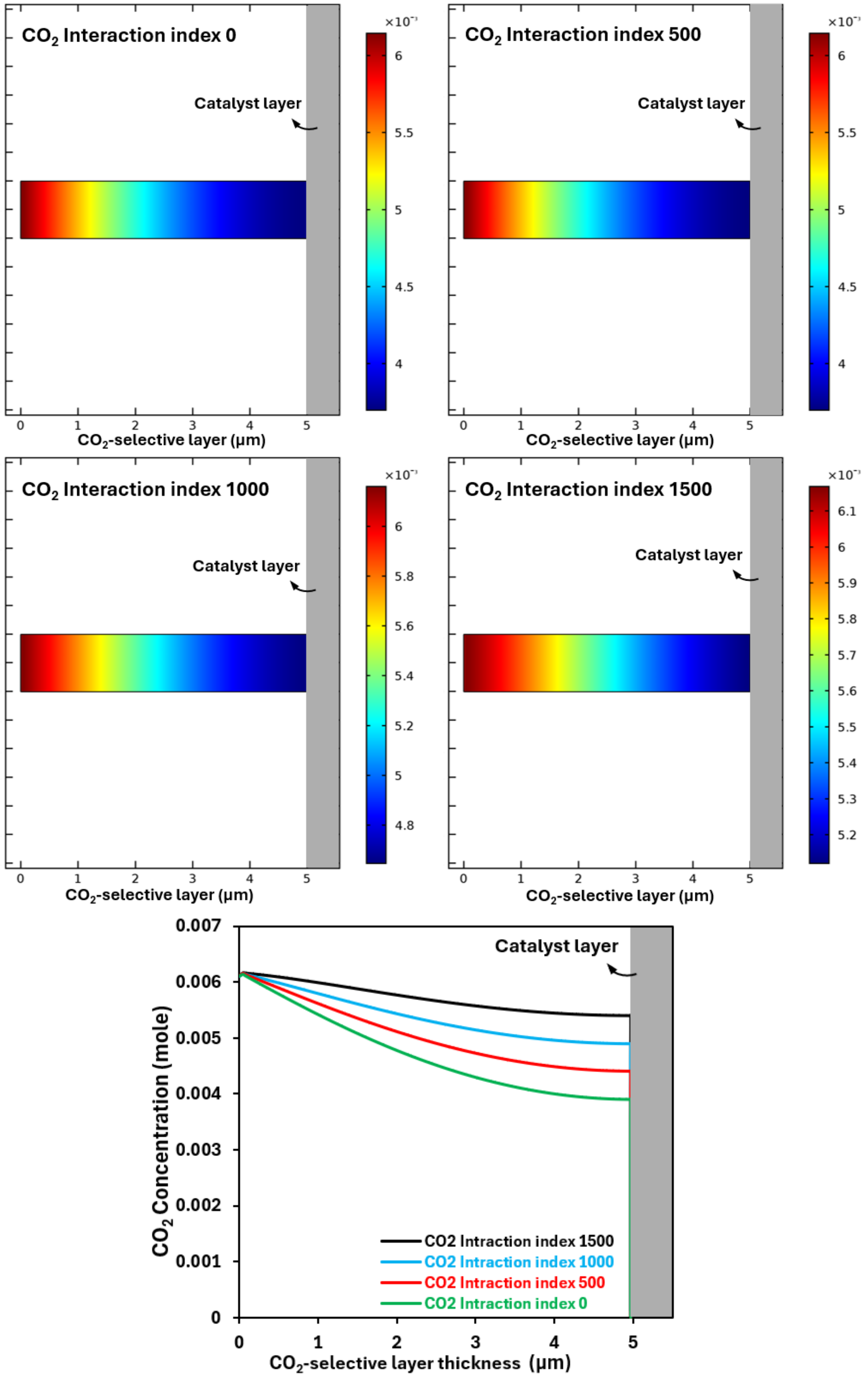

Fig. S9. COMSOL simulation of CO_2 concentration contours CO_2 in the CO_2-selective layer with different CO_2 interaction index used in the model. Higher index indicates higher affinity between the layer and CO_2 with more IL loading, and CO_2 concentration reduction in the layer.

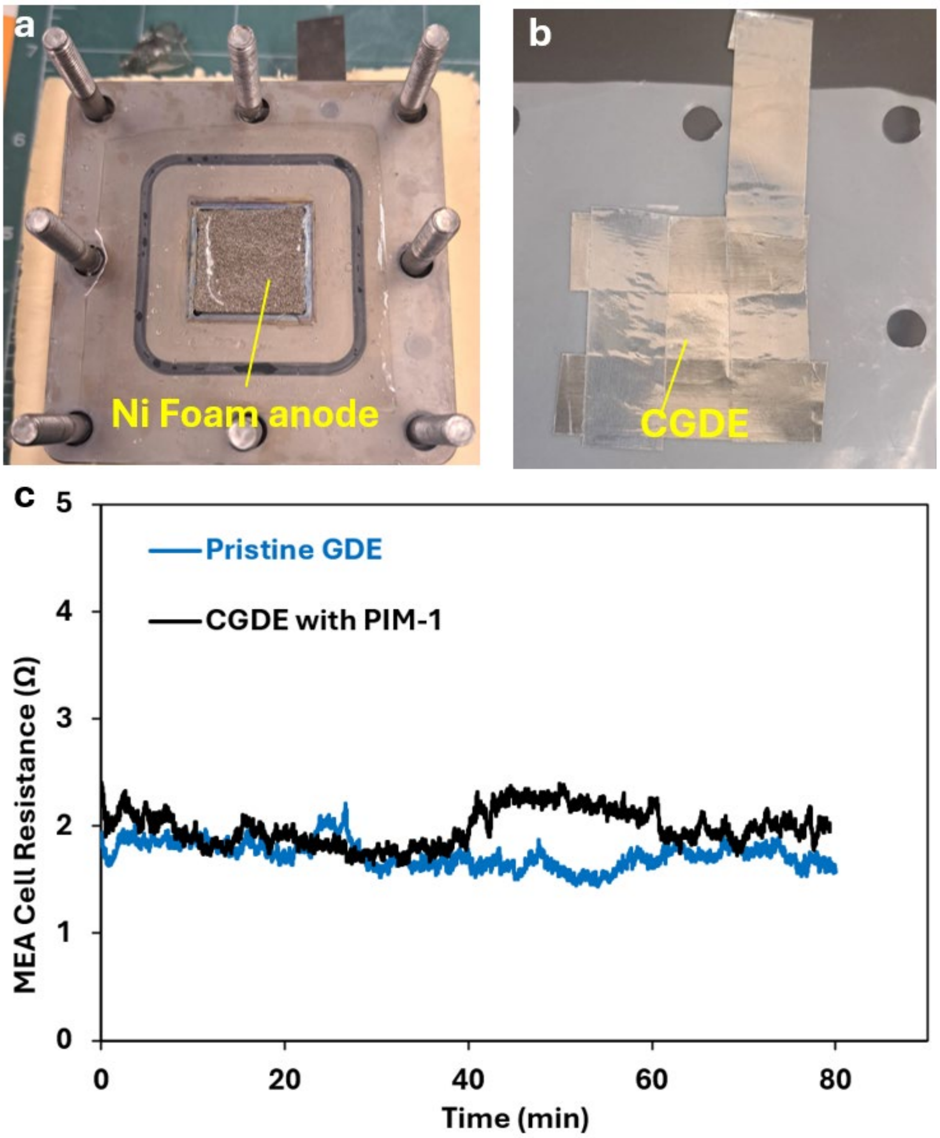

Fig. S10. The images of the a) Ni anode and b) CGDE as the cathode used in the MEA cell; c) The MEA cell resistance for the pristine GDE with Ag catalyst layer and CGDE with PIM-1 layer. No significant resistance comes from current collection from the Ag catalyst layer, therefore not influenced by the interlayer underneath. Aluminum tape was used for current collection and covered with non-conductive tape to prevent direct contact with the AEM.

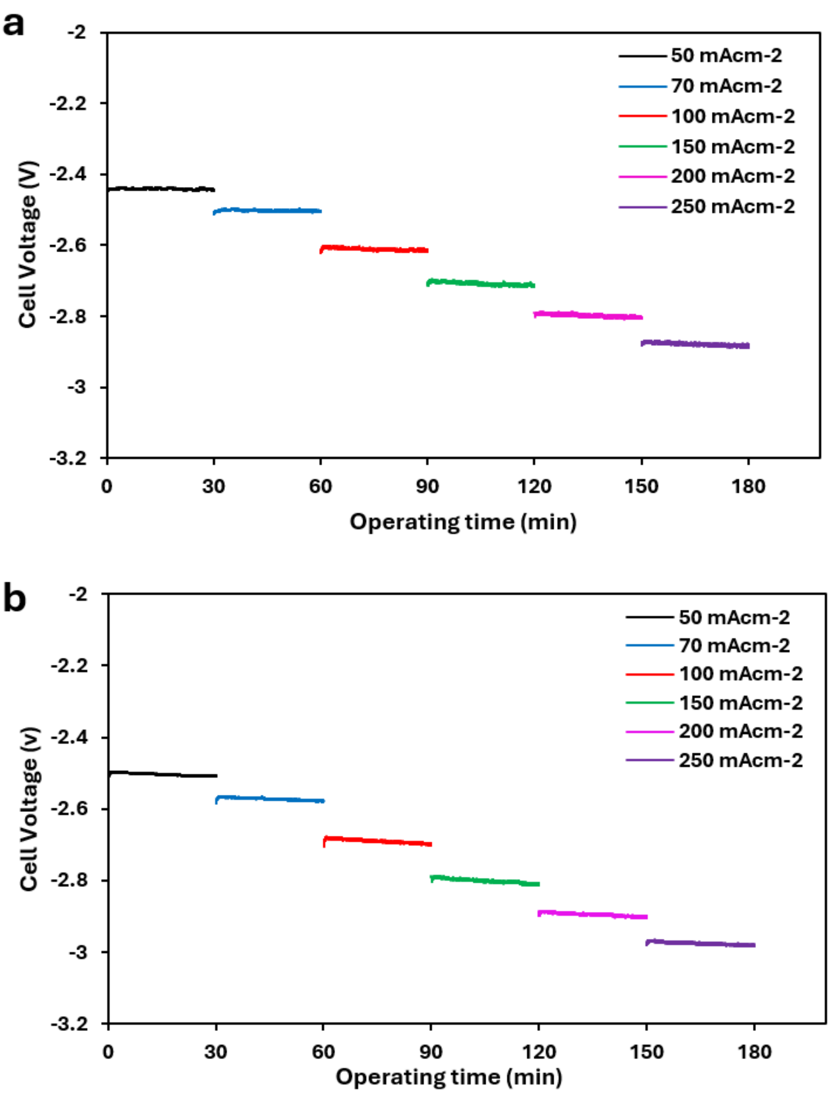

Fig. S11. The contact angle measurement for a) the PTFE substrate without coating and after coating with b) PIM-1 and c) PIM/20wt%[Emim][BF_4].

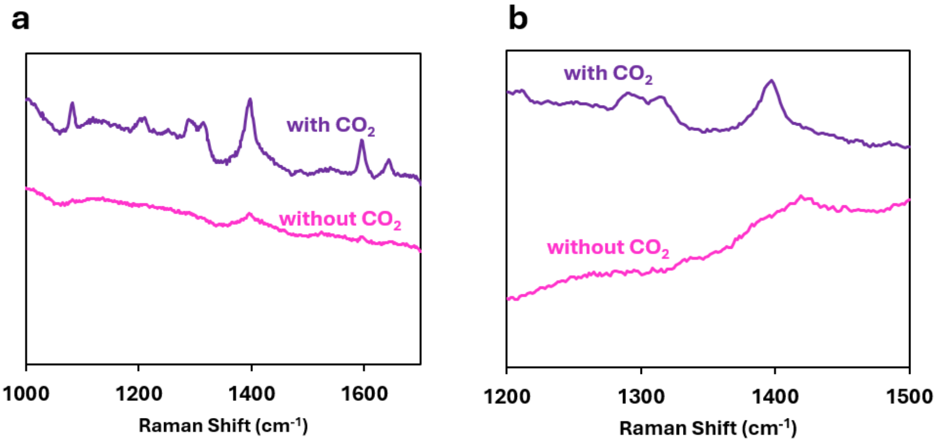

Fig. S12. The Raman spectra of key shifts in a) PIM-1 and b) [Emim][BF_4] in presence of CO_2 and Ar. The spectra in CO_2 saturated atmosphere indicates evident interaction between both PIM-1 and [Emim][BF_4] with CO_2.

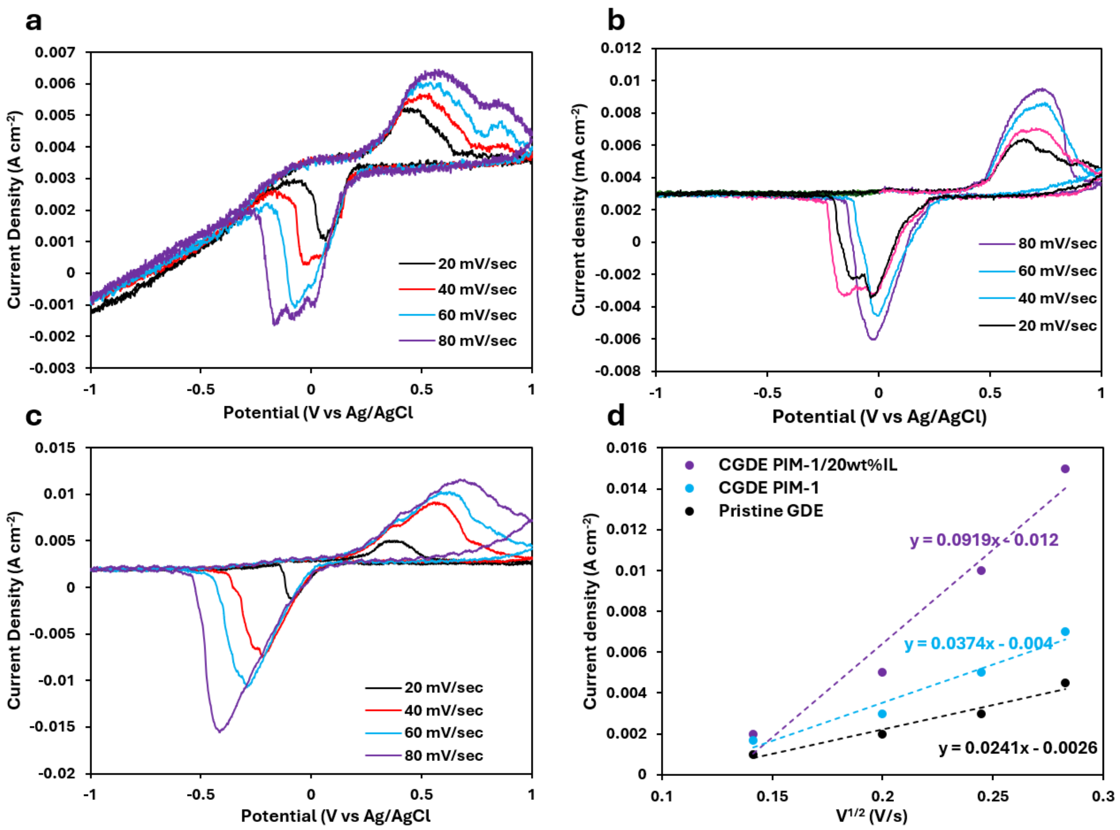

Fig. S13. CVs of **a)** Pristine GDE without any interlayer, **b)** with pure PIM-1 interlayer, and **c)** with PIM-1/20wt% [Emim][BF_4] layer from 20 to 80 mV/s; d) linear relations of current versus $V^{0.5}$ indicating the increase in the electroactive surface area according to the Randles–Ševčík relationship $I_p = (2.69\times105)\times n^{3/2}AD^{1/2}Cv^{1/2}$. I_p is peak current, A is the electroactive surface area.

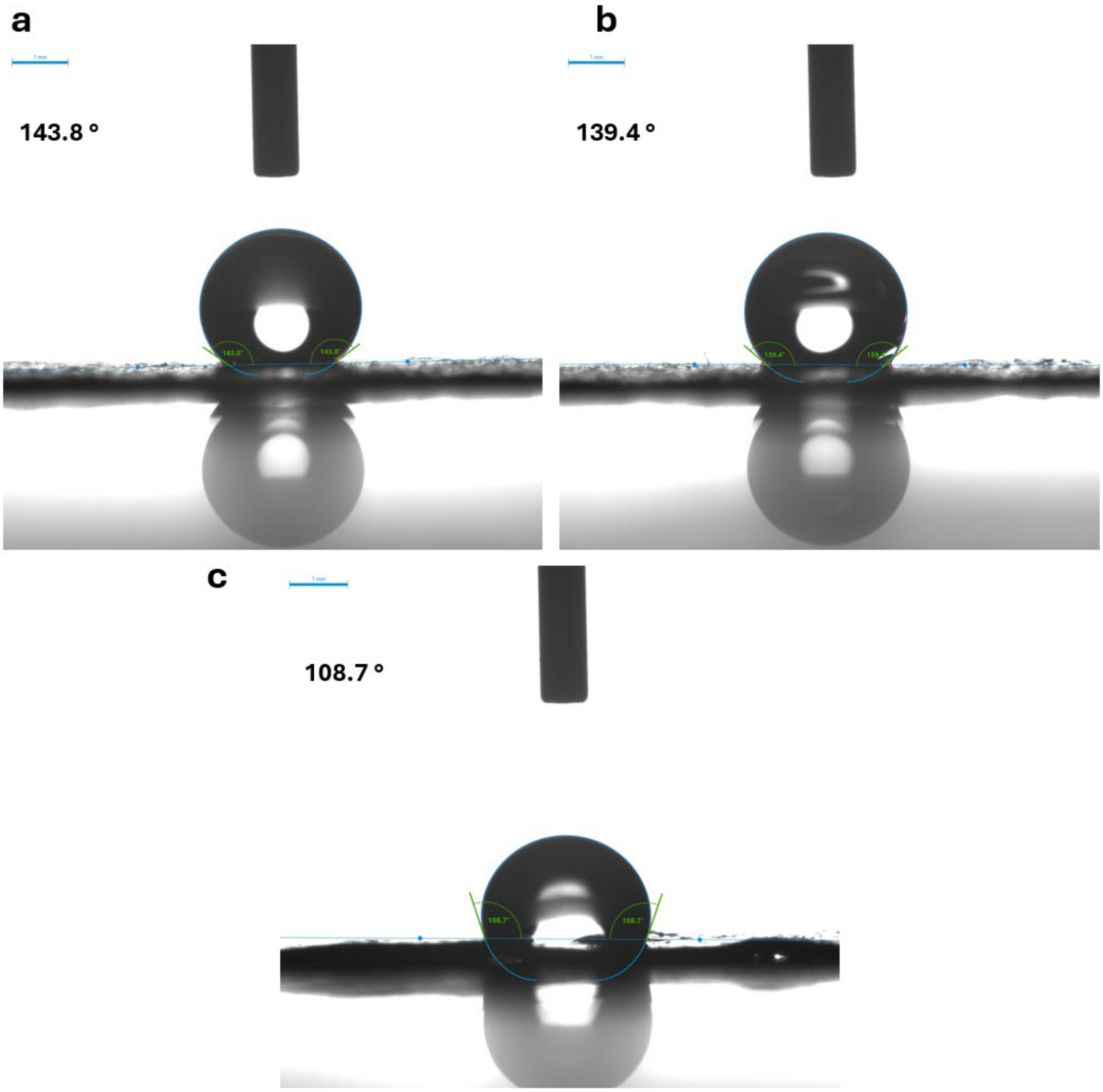

Fig. S14. The contact angle measurement for a) the PTFE substrate without coating and after coating with b) PIM-1 and c) PIM/20wt%[Emim][BF_4].

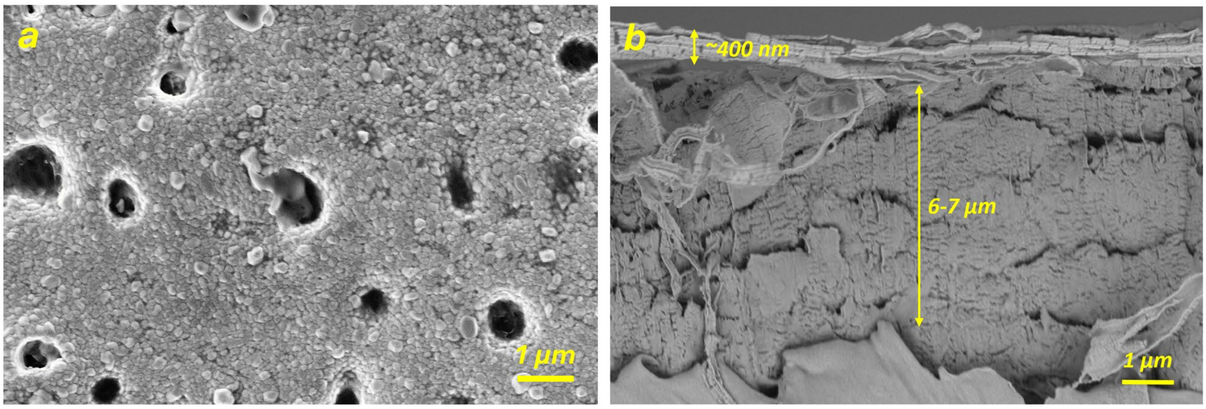

Fig. S15. a) The surface and b) cross-sectional SEM images of CGDE with PIM-1/20wt% [Emim][BF_4] selective layer after the stability test. No significant structural deformation was observed after the stability test (Ag catalyst layer (400 nm) and PIM-1/20wt% [Emim][BF_4] layer are visible).

Table S1. *Comparison of the performance achieved in this study with other relevant works on electrochemical reduction of dilute CO_2 to CO*

Catalyst	Reactor type	O_2 content	CO_2 content	Potential or cell voltage	Current density (mA cm^{-2})	FE_{CO} (%)	Ref
NiCu alloy	Flow cell	0	2-10 CO_2 % in N_2 balance	-0.33 to -0.56 V vs RHE	-25 to -50	81-98%	[4]
CALF-20 modified Ag	Flow cell, MEA	4%	10 % CO_2 in N_2 balance	-1.4 V vs RHE	-50	70-75	[5]
Co-Tpy-C (Co-N4)	Flow cell	5%	15% CO_2, N_2 balance	-0.6 V vs RHE	-24	90	[6]
MOF derived Ni-N-C	H-cell	0	15% CO_2 in Ar balance	-0.75 V vs RHE	-2.9	>90	[7]
Ni-N/C	MEA	0	10 % CO_2 in N_2 balance	-3 V	-113.6	92	[8]
Au nanoclusters	Flow cell	0	10 % CO_2 in N_2 balance	-0.36 V vs RHE	-20	90	[9]
CoPc+P4VP	MEA	0	10 % CO_2 in N_2 balance	2.75 V	-100	>95	[10]
Commercial Ag NPs	Flow cell	0	10 % CO_2 in N_2 balance	3 V	-29	80	[11]
Commercial Ag NPs	Flow cell	0	20 % CO_2 in N_2 balance	-2.71 V vs Ag/AgCl	-65	65	[12]
atomically dispersed Ni-N-C catalysts with nanoconfined ionic liquids	H-cell	0	10 % CO_2 in N_2 balance	-0.9 V vs RHE	-4.91	89.1	[13]
Ag_{12}bpy-NH_2	Flow cell & MEA	0	15 % CO_2 in N_2 balance	−2.3 V	-120	96	[14]
Ag sputtered on carbon and PTFE GDL	Flow cell	4%	15 % CO_2 in N_2 balance	-1.5 V vs RHE	-100	60	[15, 16]
Imine-Nitrogen-Doped Carbon Nanotubes	H-cell	8%	15 % CO_2 in N_2 balance	-0.83 V vs RHE	-13.8	70	[17]
CoPc/CNT with Aniline/polymer layer	Flow cell	5%	95% CO_2	3.4 V	-35	80	[18, 19]
single-atom Ni loaded on nitrogen-doped carbon	H-cell	0	20 % CO_2 in N_2 balance	-0.8 V vs RHE	-18	93	[20]
Ag	H-cell	20%	80 % CO_2	-0.7 vs. RHE	-8.2	8.8	[21]
Ag on polymer GDL	Flow cell	12%	92% CO_2	-0.88 vs. RHE	-50	40	[22]
Amine-functionalized silver	MEA	4%	15% CO_2 in N_2 balance	2.8 V	-50	78	[23]
Composite GDE with PIM-1/[Emim][Bf4] selective layer and Ag catalyst	MEA	5%	15% CO_2 in N_2 balance	2.75-2.85 V	-100 to -150	65-72%	This work

Multiphysics simulation by COMSOL

CFD modeling using COMSOL Multiphysics was employed to simulate gas transport through the membrane and catalyst [5]. A 2D model comprising two distinct sections (membrane and catalyst) was developed to investigate the concentration profiles of CO_2, N_2, and O_2.

Geometry

Two rectangles were defined in the geometry: one representing the membrane with a width of 5 μm, and the other representing the catalyst with a width of 400 nm.

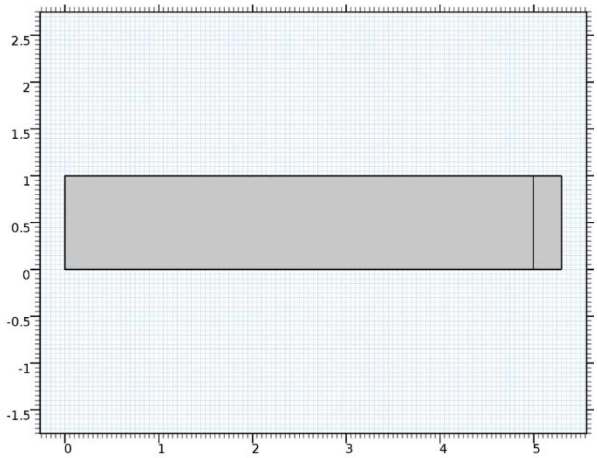

Physics

1. **Membrane:**

Transport in diluted species has been chosen for the membrane section as the primary physics model.

$$\frac{\partial c_i}{\partial t} + \nabla \cdot (-D_i \nabla c_i) + u \cdot \nabla c_i = R_i$$

$$N_i = -D_i \nabla c_i + u c_i$$

In this model the following assumptions have been made:

1. U=0 (diffusion only, no convection term)
2. R_i= Special interaction with the membrane (Gas Interaction Term, GIT). This term has been assumed to be zero for O_2 and N_2 since these components don't have any interaction with the membrane.
3. Stationary study has been selected so the $\frac{\partial C_i}{\partial t}$ is zero.

Based on the above assumptions the model can be simplified to:

$$\nabla . (-D_{i,eff} \nabla C_i) = GIT$$

Where Ci is gases concentration in the membrane, C-CO_2, C-N_2, and C-O_2

The effective diffusion coefficient has been calculated using the Bruggeman model:

$$D_{i,eff}^{MK} = \epsilon^{1.5} D_i^{MK}$$

$$D_i^{MK} = \left(\frac{1}{D_i^M} + \frac{1}{D_i^K}\right)^{-1}$$

$$D_i^M = \frac{1-\omega_i}{\sum \frac{x_i}{D_{iK}}}$$

$$D_i^K = \frac{2}{3} r_p \sqrt{\frac{8RT}{\pi M_i}}$$

r_p: pore size of medium (m)

ε: mediums gaseous volume fraction

ω_i: Mass fraction of components

Boundary condition:

- Inlet Concentration: The inlet concentration has been calculated using the molar percentage for each component and the inlet pressure:

$$C_{0i} = \frac{x_i P}{RT}$$

Where:

x_i: mole fraction (CO_2:0.15, N_2:0.8 and O_2:0.05)

P: Inlet Pressure 111.3 kPa,

T: Temperature 298 K

- No flux on the above and bottom surfaces

2. Interface:

A general partial differential equation (PDE) was employed to compute adsorption concentration at the interface (C_{si}) using the adsorption term of RA.

$$RA = k_m a_m (H_i P_i - C_{si})$$

$$k_m = \frac{D_{i,aq}}{r_p(1-\sqrt{1-S_{m)}}}$$

Where:

a_m: The available specific surface area in the medium

P_i: Each component's partial pressure,

H_i: Henry's constant, which is 34 mM atm^{-1} for CO_2, 1.3 mM atm^{-1} for O_2, and 6.4×10^{-6} mM Pa^{-1} for N_2.

S_m: The saturation of the medium (0.85)

$D_{i,aq}$: The diffusion coefficient of species i at infinite dilution (1.91× 10^{-9} m^2/s for CO_2 , 2.1×10^{-9} m^2/s for O_2 and 2×10^{-9} m^2/s for N_2)

3. **Catalyst:**

Transport in porous media was utilized for the catalyst section.

$P_{1,i}\frac{\partial ci}{\partial t}$+$P_{2,i}$+∇·$\Gamma_i$=$R_i$+$S_i$

$P_{1,i}$=ε_p

$P_{2,i}$=$c_i\frac{\partial \varepsilon p}{\partial t}$

N_i=Γ_i=-$D_{e,i}\nabla_{ci}$

The diffusion was only investigated in this catalyst. No adsorption, reaction, or convection terms have been considered. So, the equation can be simplified to:

$$\nabla.\left(-D_{ej}\nabla C_{ei}\right) = 0$$

Where:

Dej: is the diffusion coefficient of gases in the catalyst using the Bruggeman model.

Cei: gases concentration in the catalyst, Ce-N_2, Ce-CO_2, and Ce-O_2

Boundary condition:

- Concentration at the outlet: zero for CO_2, Since CO_2 consumes in catalyst,
- Flux at the outlet: zero flux for O_2 and N_2
- Concentration at the interface: C_{si}
- No flux in the above and bottom points

Study

The stationary study has been applied.

Mesh

An extremely fine mesh size was employed in the simulation.

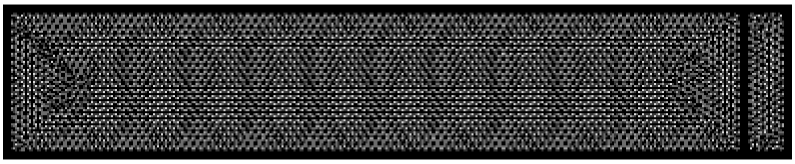

Cost estimation calculations for segregated and integrated processes

This part presents a brief cost estimation analysis of carbon monoxide (CO) production from CO_2 electroreduction (ECO_2R). The goal is to calculate the minimum selling price (MSP) of CO for three configurations: a conventional separated system, an integrated capture-conversion system within GDE with the experimental results, and an optimistic integrated system with improved efficiency. The analysis includes electricity costs, capital and operating expenses, CO_2 handling costs (capture, compression, and transport), and product separation, based on the information found in the literature with focus on technoeconomic analysis.

Main Parameters

Product: 1 metric ton of CO (1,000,000 g)
Molar mass of CO: 28.01 g/mol
Faraday constant (F): 96,485 C/mol
Electricity price: $0.07/kWh
Electrode area: 1 m^2
Cell voltage: 2.8 V
FE of CO: 80% and 65%
Current density: 200 and 150 mA cm^{-2}
Project lifetime: 10 years
Discount rate: 10%
Capital Recovery Factor (CRF): 0.1

Electrochemical Calculations

Moles of CO:
mol_CO = 1,000,000 / 28.01 ≈ 35,702 mol
Total Charge (Q):
Q = mol_CO × 2 × F ≈ 6.89×10^9 C
Energy (E):
E = Q × V
Energy (kWh) = E / 3.6×10^6
Adjusted Energy for Faradaic Efficiency (FE):
Energy_adj = Energy (kWh) / FE
Electricity Cost:
Cost_electricity = Energy_adj × electricity price

Capital Expenditure (CAPEX) [24]

Electrolyzer CAPEX is amortized over time using:
CAPEX_per_tCO = (CAPEX_per_kW × CRF) / (8760 / Energy_adj)

Cost Components by Scenario ($/tCO)

Component	Separated	Integrated	Optimistic
Electricity Cost	478.39	548.39	225.62
Product Separation [25, 26]	30.0	100.0	40.0
Non-electric OPEX [27]	50.0	50.0	50.0
Electrolyzer CAPEX	81.62	112.46	29.87
CO_2 Capture (CAPEX+OPEX) [28-32]	180	0.0	0.0
CO_2 Compression (CAPEX+OPEX) [33]	30	0.0	0.0
CO_2 Transport (CAPEX+OPEX) [33]	200	0.0	0.0
Total MSP_CO	1011.02	766.39	408.03

Scenario Descriptions

Separated: Includes standalone CO_2 capture, compression, and 1000 km transport of 5Mt of CO_2; electrolysis at 2.8 V, 80% FE, 200 mA cm^{-2}.

Integrated: Assumes capture and conversion are integrated; no compression/transport; same electrolysis setup; higher product separation cost and additional electrode layer cost. For the integrated design, electrolyzer metrics are usually lower than separated design due to the existence of N_2/O_2 in the stream, and the same FE cannot be achieved in real conditions. Herein, we considered 65% FE, 150 mA cm^{-2}.

Optimistic: Assumes advanced system operating at 1.6 V, 95% FE, 200 mA cm^{-2}; lower separation cost; includes additional electrode cost [34].

Extra Electrode Cost Estimation [35-37]

An extra layer on the electrode (5 μm thick, 1 g/cm^3 density, $100/g material cost at large scale) over 1 m^2 area adds 5 g per ton of CO. This results in a $500 cost per ton. Assuming this is amortized over 100 tons of CO, we apply a roughly $5/t$_{CO}$ adjustment to the integrated and optimistic scenarios.

Supporting Information References

[1] S. D. Bazhenov, I. L. Borisov, D. S. Bakhtin, A. N. Rybakova, V. S. Khotimskiy, S. P. Molchanov, V. V. Volkov, *Green Energy & Environment* **2016**, *1*, 235-245.
[2] F. Almansour, M. Alberto, A. B. Foster, S. Mohsenpour, P. M. Budd, P. Gorgojo, *Journal of Materials Chemistry A* **2022**, *10*, 23341-23351.
[3] A. Senocrate, F. Bernasconi, D. Rentsch, K. Kraft, M. Trottmann, A. Wichser, D. Bleiner, C. Battaglia, *Acs Appl Energ Mater* **2022**, *5*, 14504-14512.
[4] M. A. A. Mahbub, D. Das, X. Wang, G. Lu, M. Muhler, W. Schuhmann, *Angewandte Chemie* **2025**, *64*, e202419775.
[5] T. Al-Attas, S. K. Nabil, A. S. Zeraati, H. S. Shiran, T. Alkayyali, M. Zargartalebi, T. Tran, N. N. Marei, M. A. A. Bari, H. Q. Lin, S. Roy, P. M. Ajayan, D. Sinton, G. Shimizu, M. G. Kibria, *Acs Energy Letters* **2022**, *8*, 107-115.
[6] P. Hou, W. Song, X. Wang, Z. Hu, P. Kang, *Small* **2020**, *16*, e2001896.
[7] L. Jiao, W. Yang, G. Wan, R. Zhang, X. Zheng, H. Zhou, S. H. Yu, H. L. Jiang, *Angewandte Chemie* **2020**, *59*, 20589-20595.
[8] D. Kim, W. Choi, H. W. Lee, S. Y. Lee, Y. Choi, D. K. Lee, W. Kim, J. Na, U. Lee, Y. J. Hwang, D. H. Won, *ACS Energy Letters* **2021**, *6*, 3488-3495.
[9] B. Kim, H. Seong, J. T. Song, K. Kwak, H. Song, Y. C. Tan, G. Park, D. Lee, J. Oh, *ACS Energy Lett.* **2019**, *5*, 749-757.
[10] B. Chen, Y. Rong, X. Li, J. Sang, P. Wei, Q. An, D. Gao, G. Wang, *ACS Energy Letters* **2024**, *9*, 911-918.
[11] B. Kim, S. Ma, H.-R. Molly Jhong, P. J. A. Kenis, *Electrochimica Acta* **2015**, *166*, 271-276.
[12] S. Van Daele, L. Hintjens, J. Van den Hoek, S. Neukermans, N. Daems, J. Hereijgers, T. Breugelmans, *Journal of CO2 Utilization* **2022**, *65*, 102210.
[13] Q. Sun, Y. Zhao, W. Ren, C. Zhao, *Applied Catalysis B: Environmental* **2022**, *304*, 120963.
[14] Y. Y. Liu, J. R. Huang, H. L. Zhu, P. Q. Liao, X. M. Chen, *Angewandte Chemie* **2023**, *62*, e202311265.
[15] S. Van Daele, L. Hintjens, D. Choukroun, N. Daems, J. Hereijgers, T. Breugelmans, *Chem Catalysis* **2025**, 101353.
[16] S. Van Daele, L. Hintjens, S. Hoekx, B. Bohlen, S. Neukermans, N. Daems, J. Hereijgers, T. Breugelmans, *Applied Catalysis B: Environmental* **2024**, *341*, 123345.
[17] H. Shi, H. Pan, Y. Cheng, S. Lu, P. Kang, *Chemelectrochem* **2021**, *8*, 1792-1797.
[18] X. Lu, Z. Jiang, X. Yuan, Y. Wu, R. Malpass-Evans, Y. Zhong, Y. Liang, N. B. McKeown, H. Wang, *Sci Bull (Beijing)* **2019**, *64*, 1890-1895.
[19] P. Li, X. Lu, Z. Wu, Y. Wu, R. Malpass-Evans, N. B. McKeown, X. Sun, H. Wang, *Angewandte Chemie* **2020**, *59*, 10918-10923.
[20] B. Nie, X.-W. Xiong, X.-D. Xu, Y.-S. Cheng, D. Yu, Y.-S. Kang, K.-L. Wu, F.-H. Wu, G. Yuan, X.-W. Wei, *Inorganic Chemistry Communications* **2023**, *153*, 110876.
[21] C. Li, H. Xiong, M. He, B. Xu, Q. Lu, *ACS Catalysis* **2021**, *11*, 12029-12037.
[22] A. Anzai, M. Higashi, M. Yamauchi, *Chem Commun (Camb)* **2023**, *59*, 11188-11191.
[23] Z. Liu, T. Yan, H. Shi, H. Pan, P. Kang, *Applied Catalysis B: Environmental* **2024**, *343*, 123456.
[24] M. H. Barecka, J. W. Ager, A. A. Lapkin, *STAR Protoc* **2021**, *2*, 100889.
[25] M. Jouny, W. Luc, F. Jiao, *Industrial & Engineering Chemistry Research* **2018**, *57*, 2165-2177.
[26] E. National Academies of Sciences, Medicine, *Negative Emissions Technologies and Reliable Sequestration: A Research Agenda*, The National Academies Press, Washington, DC, **2019**.
[27] Z. Huang, R. G. Grim, J. A. Schaidle, L. Tao, *Energy & Environmental Science* **2021**, *14*, 3664-3678.
[28] **IEA (2021), Carbon capture, utilisation and storage: the opportunity in Southeast Asia, IEA, Paris https://www.iea.org/reports/carbon-capture-utilisation-and-storage-the-opportunity-in-southeast-asia, Licence: CC BY 4.0.**
[29] T.-E. A. o. S.-S. C. C. f. I. a. P. S. 'IEAGHG, 2024-03, March 2024.
[30] D. W. Keith, G. Holmes, D. St. Angelo, K. Heidel, *Joule* **2018**, *2*, 1573-1594.
[31] P. Friedlingstein, M. O'Sullivan, M. W. Jones, R. M. Andrew, L. Gregor, J. Hauck, C. Le Quéré, I. T. Luijkx, A. Olsen, G. P. Peters, W. Peters, J. Pongratz, C. Schwingshackl, S. Sitch, J. G.

Canadell, P. Ciais, R. B. Jackson, S. R. Alin, R. Alkama, A. Arneth, V. K. Arora, N. R. Bates, M. Becker, N. Bellouin, H. C. Bittig, L. Bopp, F. Chevallier, L. P. Chini, M. Cronin, W. Evans, S. Falk, R. A. Feely, T. Gasser, M. Gehlen, T. Gkritzalis, L. Gloege, G. Grassi, N. Gruber, Ö. Gürses, I. Harris, M. Hefner, R. A. Houghton, G. C. Hurtt, Y. Iida, T. Ilyina, A. K. Jain, A. Jersild, K. Kadono, E. Kato, D. Kennedy, K. Klein Goldewijk, J. Knauer, J. I. Korsbakken, P. Landschützer, N. Lefèvre, K. Lindsay, J. Liu, Z. Liu, G. Marland, N. Mayot, M. J. McGrath, N. Metzl, N. M. Monacci, D. R. Munro, S.-I. Nakaoka, Y. Niwa, K. O'Brien, T. Ono, P. I. Palmer, N. Pan, D. Pierrot, K. Pocock, B. Poulter, L. Resplandy, E. Robertson, C. Rödenbeck, C. Rodriguez, T. M. Rosan, J. Schwinger, R. Séférian, J. D. Shutler, I. Skjelvan, T. Steinhoff, Q. Sun, A. J. Sutton, C. Sweeney, S. Takao, T. Tanhua, P. P. Tans, X. Tian, H. Tian, B. Tilbrook, H. Tsujino, F. Tubiello, G. R. van der Werf, A. P. Walker, R. Wanninkhof, C. Whitehead, A. Willstrand Wranne, R. Wright, et al., *Earth System Science Data* **2022**, *14*, 4811-4900.

[32] D. Y. C. Leung, G. Caramanna, M. M. Maroto-Valer, *Renewable and Sustainable Energy Reviews* **2014**, *39*, 426-443.

[33] E. Smith, J. Morris, H. Kheshgi, G. Teletzke, H. Herzog, S. Paltsev, *International Journal of Greenhouse Gas Control* **2021**, *109*, 103367.

[34] M. Li, E. Irtem, H. P. Iglesias van Montfort, M. Abdinejad, T. Burdyny, *Nat Commun* **2022**, *13*, 5398.

[35] A. Ayyaz, A. B. Foster, L. Cseri, G. Szekely, P. M. Budd, *Acs Sustain Chem Eng* **2025**, *13*, 2784-2792.

[36] D. A. Gkika, V. Filiz, S. Rangou, G. Z. Kyzas, A. C. Mitromicronpoulos, *Membranes (Basel)* **2022**, *12*.

[37] W. H. D. Goh, H. S. Lau, W. F. Yong, *Sci Total Environ* **2023**, *892*, 164582.
